# Linking species local trends from assemblage monitoring to global extinction risk

**DOI:** 10.1038/s41467-026-74132-7

**Published:** 2026-06-23

**Authors:** Laura H. Antão, Faye Moyes, Maria Dornelas, Shane A. Blowes, Brian J. McGill, Cher F. Y. Chow, Ada Fontrodona-Eslava, Anne E. Magurran, Nicholas J. Gotelli

**Affiliations:** 1 Research Centre for Ecological Change, Faculty of Biological and Environmental Sciences, University of Helsinki, Helsinki, Finland; 2 Department of Biology, University of Turku, Turku, Finland; 3 Centre for Biological Diversity, University of St Andrews, Fife, UK; 4 Scottish Oceans Institute, University of St Andrews, Fife, UK; 5 Guia Marine Laboratory, MARE, Faculdade de Ciências da Universidade de Lisboa, Cascais, Portugal; 6 Leverhulme Centre for Anthropocene Biodiversity, Department of Biology, University of York, Wentworth Way, York UK; 7 German Centre for Integrative Biodiversity Research (iDiv), Halle-Jena-Leipzig, Germany; 8 Department of Computer Sciences, Martin Luther University, Halle-Wittenberg, Germany; 9 School of Biology and Ecology and Mitchell Center for Sustainability Solutions, University of Maine, Orono, ME USA; 10 Cellular and Organismic Networks, Faculty of Biology, Ludwig-Maximilians-Universität München, Planegg-Martinsried, Germany; 11 Department of Biology, University of Vermont, Burlington, VT USA

**Keywords:** Ecosystem ecology, Conservation biology, Biodiversity

## Abstract

While biodiversity is being reshaped across the globe, extinction risk assessments are lacking for most species, and a major challenge remains in understanding whether global threat status aligns with local population trends. Here, we assess whether population temporal prevalence trends are consistent with a species’ global extinction risk, using over 60,000 populations of 2362 species across 978 marine and terrestrial assemblages (sampled for at least 20 years, mostly from temperate regions). We assign each population to one of five categories of temporal prevalence dynamics, and retrieve each species’ extinction risk from the International Union for Conservation of Nature (IUCN) Red List. Fewer than 10% of local populations show consistent increasing or decreasing prevalence over time, with most exhibiting random patterns of temporal change, especially marine populations. Overall, higher extinction risk is associated with a higher frequency of decreasing local prevalence, and vice-versa for increasing prevalence, against a backdrop of complex links between extinction risk and local temporal dynamics. Our results suggest that directional changes in species local prevalence could be harbingers of future changes in global threat status, and highlight how leveraging assemblage monitoring data can aid conservation efforts and extinction assessments.

## Abstract (web summary)

Most species lack extinction risk assessments. This study shows decreasing local prevalence within assemblages correlates with extinction risk, with complex links between extinction risk and local temporal dynamics, highlighting the value of assemblage monitoring.

## Introduction

As global environmental change continues to accelerate, extinction risks are rising^1–4^ and assemblages are being reorganised across taxa, biomes and realms^5–8^. A better understanding of the processes that underpin such biodiversity changes is crucial for improving predictions and conservation strategies. Two complementary approaches to quantifying biodiversity change are species-level extinction assessments and assemblage-level biodiversity analyses. The first of these is often based on the International Union for Conservation of Nature (IUCN) Red List of Threatened Species, which assesses the extinction risk for over 160,000 species based on a set of five quantitative criteria related to species’ population size, trend, and geographic range^9–11^. Currently, over 48,000 species are classified as threatened with extinction (28% of those assessed), with high frequencies of at-risk species for cycads (71%), amphibians (41%), and sharks and rays (38%). The IUCN Red List classification is a fundamental framework for conservation and biodiversity research, e.g., informing policy decisions and contributing key indicators to the Kunming-Montreal Global Biodiversity Framework (https://www.cbd.int/gbf) and the Intergovernmental Science-Policy Platform on Biodiversity and Ecosystem Services (IPBES) reporting^1^. However, taxonomic biases, lack of data and inadequate (re)assessments hamper the urgent need for accurate and comprehensive estimation of species extinction risk^11–13^. This can lead to uncertainty in the proportions of species currently threatened, due e.g., to misclassification or underestimation of risk, as well as inconsistency with other estimates of extinction risk^11,12,14–18^.

In contrast to species-level extinction assessments, assemblage-level studies use long-term monitoring data to quantify temporal change in multiple biodiversity metrics^19–22^. Systematic analyses of different assemblage-level monitoring data have revealed approximately balanced positive and negative trends in species richness, total abundance^6,8,23,24^, and population trajectories^25–28^, alongside strong evidence for widespread changes in species composition^6,8,23,29,30^. Due to their different focus and aims, species-level extinction assessments and assemblage-level biodiversity analyses are pursued largely in isolation, and sometimes reveal hard to reconcile patterns of change^30^ – for instance, elevated global extinction rates alongside a mix of population and diversity trends at smaller scales^6,23–25,27,31^ In addition, studies evaluating broad-scale patterns of extinction risk mostly focus on individual populations, on particular taxa or use proxies to estimate extinction risk^4,11,14,16,26,32,33^. Thus, no quantitative link has been tested between a species’ IUCN-based extinction risk and its temporal prevalence in assemblage monitoring surveys (except for a few isolated studies examining patterns of rarity, e.g., see refs. ^34,35^).

Here, we provide a systematic assessment of the relationship between the extinction status of a species and its temporal prevalence in assemblage monitoring data, rather than population-level data as usually used in IUCN extinction assessments. We use data from BioTIME^21,36^, a database of assemblage time series which includes standardised monitoring data for a range of taxonomic groups, e.g., plants, invertebrates, birds, mammals and fish. We select assemblages that have been consistently sampled for at least 20 years, yielding 2362 species in 978 marine and terrestrial assemblages mostly from temperate regions (Fig. S1). We emphasise that we do not aim to validate or test IUCN classification or assessment criteria (*c.f*.^11,14,18,32^). Rather, we aim to assess whether temporal prevalence trends of populations from long-term assemblage monitoring data reflect a signal consistent with the IUCN extinction risk categories across species and habitats.

## Results

### Patterns of temporal prevalence

**Fig. 1:**
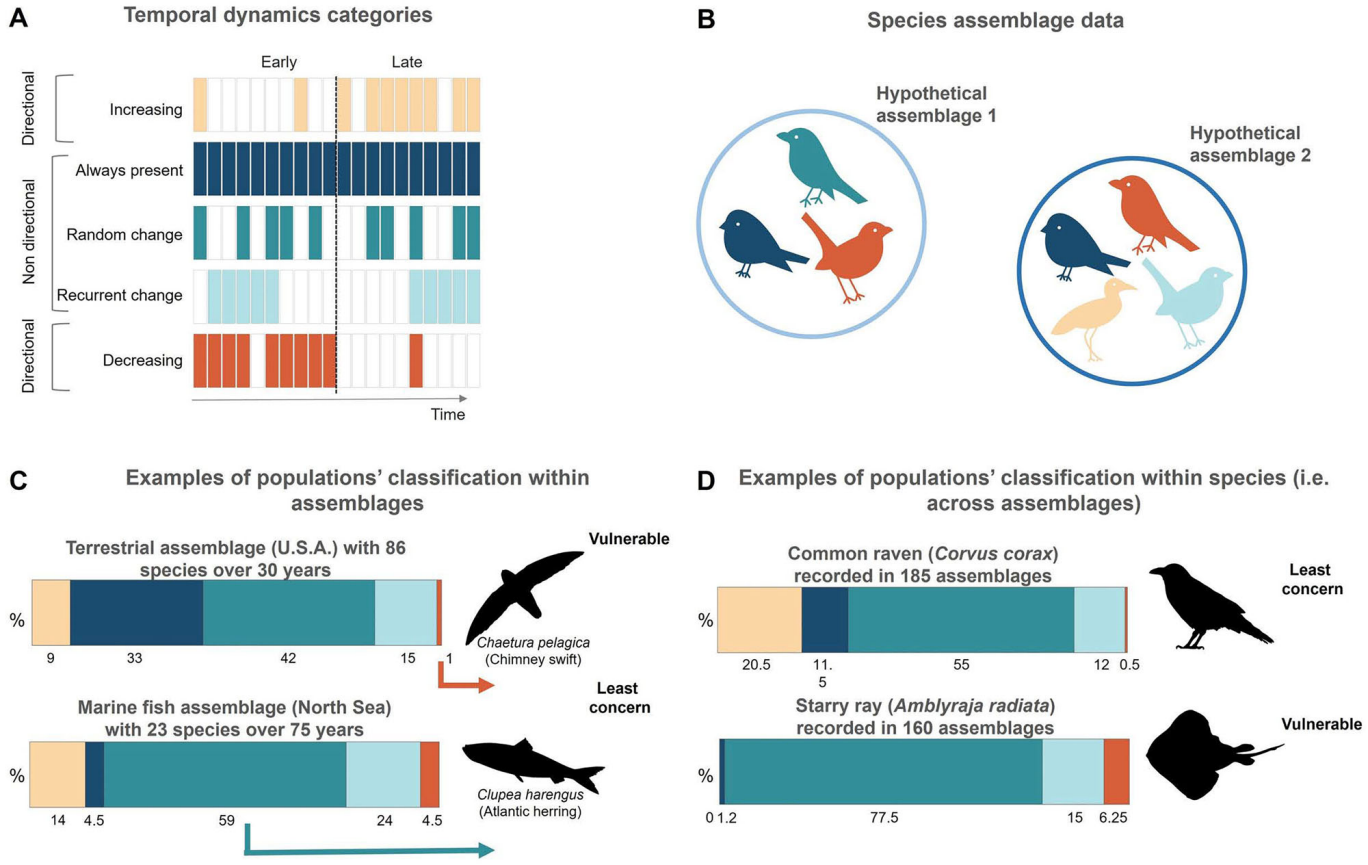
Illustration of the temporal dynamics categories and population classification. (**A**) shows the five categories of temporal dynamics used to classify each population within assemblages, with hypothetical examples in (**B**). (**C**) shows one marine and one terrestrial assemblage from our data, illustrating how the populations in each are classified into the different temporal categories, represented by the coloured bars (%). In each assemblage we highlight one species with different temporal prevalence and IUCN extinction risk: *Clupea harengus* (Least Concern) and *Chaetura pelagica* (Vulnerable). **D** shows two species in our data, the common raven (*Corvus corax*) and the starry ray (*Amblyraja radiata*), illustrating how different populations of the same species can be classified into different temporal categories among assemblages, where the coloured bars indicate the percentage of populations falling within each category across all the assemblages in our data where each species was recorded.

We first quantified patterns of temporal prevalence for the 66,209 populations of the 2362 species in our data. Temporal prevalence was quantified as the outcome of a series of statistical tests that characterise variation in species incidence over time^37^. We classified every population in each assemblage into one of five mutually exclusive categories of temporal dynamics (see Methods for details). Three of the categories represent patterns of non-directional change: always present, random change and recurrent change, which correspond to species present throughout the time series, species with a random pattern of presences and absences (no statistically detectable pattern), and species that persist and are absent during relatively long periods, respectively (Fig. 1A). The other two categories represent directional change: increasing and decreasing temporal prevalence through time (Fig. 1A). A temporal trend of decreasing prevalence in a local assemblage could be an early-warning indicator of a species at risk of regional or global extinction^4,16^. Conversely, a trend of increasing prevalence could indicate the arrival of a novel species, e.g., due to the expansion or shift of its geographic range, or potentially reflect recovery following successful conservation actions. We anticipated that most species would exhibit random patterns in their temporal prevalence, with only a small proportion of populations showing either decreasing or increasing temporal prevalence^37^. In addition, because marine and terrestrial communities can exhibit different patterns of biodiversity change^6,38,39^, we expected the overall patterns of temporal dynamics to differ between realms. To test these expectations, we fit a mixed model to the vector of proportions of the temporal categories within each assemblage as a function of realm (setting random change as the baseline category; Methods).

**Fig. 2:**
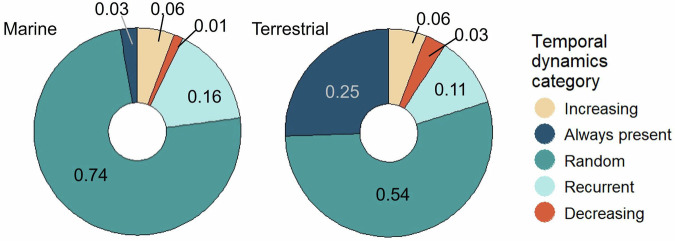
Proportion of populations classified into the five temporal dynamics categories in marine and terrestrial assemblages. The data included 2362 species with *n* = 66,209 populations (41,202 terrestrial and 25,007 marine) in 978 assemblages (481 terrestrial and 497 marine). The estimated proportion values from a mixed effect model are shown in Fig. S2 and Table S1.

Overall, the proportion of populations classified into the different temporal categories differed between realms (Fig. 2, Fig. S2, Table S1). Most populations were characterized by random change, which had a higher prevalence in the ocean (Fig. 2). The two directional categories accounted for small proportions in both realms (7% marine and 9% terrestrial), with cases of increasing prevalence being more common than decreasing prevalence (Fig. 2). Very few marine populations were always present over time ( ~ 3%), contrasting with ~25% of terrestrial populations (Fig. 2). Given this large-scale quantification of the distribution of temporal prevalence dynamics over tens of thousands of populations across taxonomic groups and spanning multiple ecosystems, our results suggest these patterns of population temporal prevalence are widespread, whereas a previous assessment had focused on temperate marine fish assemblages only^37^.

### Relationship between extinction risk and local temporal dynamics

We then assessed whether extinction risk is systematically related to the observed temporal dynamics of the local populations within assemblages. Each of the 973 species in our data with an IUCN classification was assigned to one of four extinction risk categories^10^ (see Methods; *n* = 53,235 populations; we excluded species classified as Data Deficient from our analysis). This second analysis mainly included data for vertebrates, with limited matches for plants, crustaceans and molluscs. We fitted linear mixed models to extinction risk as a function of each populations temporal dynamics category, and included an interaction term for realm to test if the effects differed between marine and terrestrial species.

**Fig. 3:**
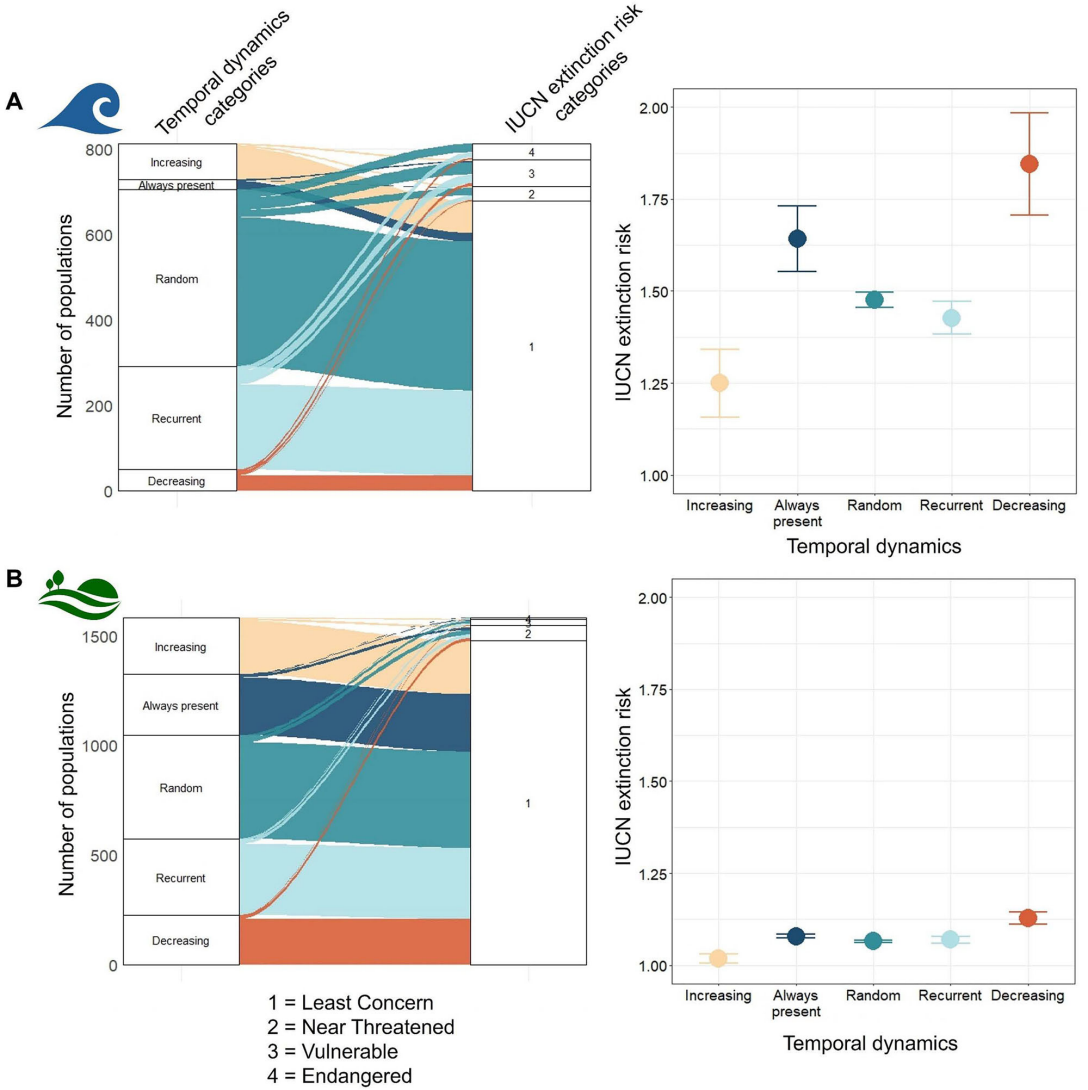
Relationship between IUCN extinction risk categories and local population temporal dynamics within assemblages. Marine (**A**) and terrestrial (**B**) species are shown separately (total *n* = 53,235 populations from 973 species). Extinction risk categories are 1 = Least Concern, 2 = Near Threatened, 3 = Vulnerable and 4 = Endangered/Critically Endangered. The left panels illustrate the empirical association between each temporal dynamics and each extinction risk category. The right panels show the estimated probabilities from the (two-sided) mixed model where extinction risk was included as a continuous variable (Table S3); the error bars represent the 95% confidence intervals around the predicted values (see Table S2 and Fig. S7 for underpinning contingency table and results of Fisher’s exact tests on the combinations between extinction risk and temporal dynamics categories). Data are presented as mean values +/− 95% confidence intervals based on the standard error (SE).

We detected a robust signal between extinction risk and local temporal dynamics (Fig. 3, Table S3). Specifically, species with higher extinction risk were more likely to have populations with decreasing prevalence, and less likely to have increasing temporal prevalence, compared to random dynamics (Fig. 3, Table S3). This signal was detected despite the complex links between species extinction risk and population temporal dynamics, as shown by the high variation in the relationship between the IUCN classification and our temporal prevalence categories, and even though most species in our data were classified as Least Concern, suggesting that extinction risk may be of low probability for the species in our data (Fig. S3, Table S2). Indeed, such a link would be hard to detect because many highly threatened species have small geographic ranges, may be less likely to occur in biodiversity monitoring surveys, and are more likely to have very low abundances. Examples of endangered species with declining local prevalence in the assemblage data include the chimney swift (*Chaetura pelagica*), the winter skate (*Leucoraja ocellata*) and the starry ray (*Amblyraja radiata)*, which may nonetheless fall into different temporal dynamics among time series of different populations (Fig. 1D). Conversely, the European starling (*Sturnus vulgaris*), the house sparrow (*Passer domesticus*), and the ring-necked pheasant (*Phasianus colchicus*) are examples of non-threatened species with increasing local prevalence in our data. Interestingly, the latter two species are considered problematic non-native species in North America^40^, despite e.g., *P. domesticus* experiencing recent declines in the UK^41^, Europe^42^, and areas of India^43^. In addition, we found a higher probability of a population being always present with increasing extinction risk compared to random dynamics, and inversely a lower probability of being characterised by recurrent change. Furthermore, these effects were overall stronger in the ocean (Fig. 3, Table S3). However, given the small number of species in high-risk categories in our data, which was particularly low for terrestrial species (Fig. S3, Table S2), we mainly focus on the overall relationship between extinction risk and temporal dynamics. Moreover, our results remained consistent with analysis designed to ensure that our results were not overly influenced by the predominance of Least Concern species in our data that used a simplified binary threat classification of threatened vs non-threatened species (see Methods), as well as for analyses restricted to temperate regions only (Figs. S2, S4–S6, Tables S1, S4–S6), which were the best represented in our data (Fig. S1).

## Discussion

Our analysis has uncovered a previously undocumented relationship between species’ IUCN extinction risk and directional change in temporal prevalence within assemblages that is detectable across ecological realms and several taxonomic groups. Overall, species with decreasing temporal prevalence were associated with a higher risk of extinction, whereas species with increasing prevalence were associated with lower extinction risk, revealing some degree of consistency between IUCN risk categories and local trends in temporal prevalence estimated from assemblage monitoring data. Moreover, we also revealed that the relationship between local temporal prevalence and extinction risk is complex. For example, populations of non-threatened or low risk species displayed a range of different temporal dynamics, including decreasing or increasing prevalence, and some populations of threatened species were found to be continuously present. It is important to recognise that our analysis focuses on individual population trajectories and that a species can occur in more than one temporal change category. For example, the pogge, *Agonus cataphractus*, a widely distributed fish, fell into three categories in our data: decreasing, random or recurrent, depending on which assemblage it was recorded in, and the commercially-important Atlantic cod (*Gadus morhua*) was always present in the Baltic Sea, while showing decreasing or random dynamics in the North Sea and the Celtic Sea, respectively. Thus, our results underline the importance of evaluating a species extinction risk across its range, and highlight the role of local conditions and assemblage dynamics in shaping population trends^44,45^. Spatial variability in species temporal trends, as well as mismatches between temporal dynamics and IUCN extinction risk classification are consistent with recent reports, including e.g., for vertebrates^4,16,26^, and butterflies^27^ and birds^46^ in North America. While this poses important challenges for global estimation of extinction risk^32^, conservation policy is often implemented at regional and national levels, where regional and local assessments are critical.

Long-term decreases in species prevalence may be harbingers of future threat status and extirpation, and potentially global extinction. The relationship between local declines and IUCN-based global extinction risk described here suggests that species exhibiting decreasing temporal dynamics may be vulnerable to local, or even global, extinction even if they have not yet been formally classified as at risk^15,16,18^. This high uncertainty in estimating the true number of threatened species skews our understanding of global biodiversity change and hampers efforts to accurately determine current rates of extinction^13,16,18,47^. On the other hand, increasing temporal prevalence was more common than decreasing trends in our data. While cases where local extinctions outnumber local colonisations have been widely reported^16,48^, there are also many instances with more balanced colonisation and extinction events^25,49^, as well as examples of a higher prevalence of populations with increasing temporal incidence compared to decreasing ones – e.g., when colonisation rates exceed extirpation rates^8,50,51^, or when range expansions outpace range contractions^52–54^.

Marine populations largely exhibited random temporal prevalence, while a large proportion of terrestrial populations were always present. This has implications for monitoring and analyses of biodiversity and extinction risk among realms, and further emphasises the importance of long-term monitoring for accurate detection of patterns of change^19–22^. Differences between marine and terrestrial ecosystems are well documented^6,55,56^, and our findings are consistent with higher dispersal^57,58^ and stronger responses to warming in the ocean^38,53,59^. The pattern of predicted extinction risk levels across temporal dynamics categories was similar in marine and terrestrial realms, but our results suggest that the magnitude of the associations differed between them. This difference could reflect a data gap, as marine species are often less well-studied than terrestrial ones^60^. Our framework thus provides a useful avenue to identify marine species of potential concern, given the large availability of biodiversity monitoring data for marine taxa.

By leveraging the potential of assemblage monitoring data, our approach provides a straightforward path for complementing and improving extinction risk assessments, with several key strengths. First, using longer time series provides more accurate predictions of trends and extinction risk^18,61,62^. Additionally, one important criticism of the IUCN methodology is the arbitrary choices of a 10-year window (or three generations) and the baseline year for estimating population reduction, which can cause classifications to vary across extinction risk categories, and lead to erroneous classifications, e.g., if a decline occurred before the focal 10-year period or due to rapid population changes going unrecognized^11,18,63^. As such, having data preceding any baseline year chosen for assessments or covering longer periods is key to more reliably estimating trends. Second, assessing a range of temporal dynamics, rather than focusing solely on declines, provides robust insight into different types of temporal trends^16,25,26,28,46,64^. Third, using assemblage monitoring data allows estimating temporal trends for many more species, thus contributing to alleviate persistent taxonomic and ecosystem biases^11,14,18^, and providing potential alternative sources of data and estimates to reduce the number of unassessed species or classified as Data Deficient, e.g. when population-level monitoring may not be available to evaluate current criteria^11,15^. Nonetheless, a few important considerations and caveats need to be acknowledged. First, and although we have analysed the largest and most comprehensive databases for both assemblage time series and global extinction risk estimation currently available, spatial and taxonomic biases are pervasive – namely towards vertebrates in IUCN^14,16^, and for fish, birds and marine invertebrates, mostly from Europe and North America in BioTIME^21^. Moreover, there was relatively little species overlap between the two sources: only 15% of the species recorded in BioTIME are included in the Red List, and only 10% of Red List species are found in BioTIME. This results from most assemblage-level monitoring programs not taking place within the small geographic ranges of many globally threatened species, and less than 8% of known species having been assessed by the IUCN Red List^14,32^. Finally, unlike many IUCN species assessments^14,18^, assemblage-level data do not focus on threatened species. Notably, amphibian and tropical species were not included in our analysis, two groups that systematically have high extinction risk and decreasing populations^4,10,16,26^. Nonetheless, tropical regions can also harbour stable populations^16^, again emphasising the need to consider a range of temporal trends, as well as variation among regions and taxa^16,65^.

Our results have direct implications for conservation and management. Assemblage-level biodiversity analyses could highlight the species and populations to be prioritised for targeted assessments and conservation actions^11^, thus shifting the focus from global blanket estimates of extinction risk to regional and local contexts^32,66^. For instance, targeting efforts towards increasing and stable populations of threatened species could yield large benefits, e.g., allow maintenance of effective population size, prevent genetic erosion^67^, and potentially alleviate extinction debts, as continuous presence may nonetheless mask abundance declines and lead to extirpations^68^. By alleviating data caveats and assessing local population changes to reveal the full range of temporal prevalence dynamics, assemblage monitoring data can elucidate the variability in species trends across their ranges, or along gradients of environmental or anthropogenic drivers, and directly contribute to improve estimation of Red List criteria related to species’ population size and trends^11,32^, as well as towards the IUCN Green List of Species^10,62^.

One paradox of the biodiversity crisis is that assemblage properties, such as species richness and total abundance, do not necessarily show directional trends, but can be accompanied by marked compositional change^6,8,69–71^. Previous work^37^ showed that species with decreasing and increasing temporal prevalence are influential in driving compositional change at the assemblage level. Assemblage-based approaches that account for the full range of temporal dynamics can therefore support informed conservation planning and improve our understanding of biodiversity change. By showing that species of conservation concern are also associated with the restructuring of the assemblages they are embedded in, our study reinforces the recognition that actions to support local populations of threatened taxa could help moderate the compositional reorganisation of local assemblages. Finally, our findings establish linkages between two approaches - species-level extinction assessments and assemblage-level biodiversity analyses - that have traditionally been pursued independently, and thus aid in reporting complex biodiversity dynamics to policy makers and managers.

Given the size of the task of quantifying biodiversity change for the entire planet, identifying ways to leverage complementary approaches and data is critical. For instance, incorporating other estimates of extinction risk, such as traits, range size, and occupancy trajectories from the fossil record^17,72,73^ and thermal biases within assemblages^74,75^ can provide urgent improvements towards a more comprehensive evaluation of extinction risk^76^. Increased cohesion between global, regional, and local scale assessments, and between biodiversity change research and conservation planning is an important challenge for both scientists and practitioners^77^. As global change continues to accelerate, more accurate estimations of which species and populations require protection is urgently needed, as is providing evidence of conservation action successes, thus aiding in effectively steering research and conservation efforts^78^. Our findings provide clear avenues to alleviate some of these challenges.

## Methods

To test for a link between IUCN-based extinction risk classifications and population temporal dynamics within assemblages, we used BioTIME, which is the largest database of assemblage time series including different taxonomic groups and biomes^21,36^. BioTIME currently includes 386 studies, with over 12 million abundance records for over 44 thousand species including plants, invertebrates, fish, birds and mammals from the marine, terrestrial and freshwater realms. We followed the procedure outlined in refs. ^6,38^ to account for the heterogeneity in spatial extent across the different studies and quantify temporal patterns using a common spatial resolution across studies and realms. Briefly, studies with large spatial extents were split into ~96 km^2^ hexagonal grid cells, which were then analysed as individual assemblage time series (for further details see refs. ^6,38^). From these spatially harmonised data, we selected marine and terrestrial time series with at least 20 (not necessarily consecutive) years of sampling and a minimum of 10 species. These criteria resulted in 978 assemblages (481 terrestrial and 497 marine) from 42 original studies, including 2362 species (818 terrestrial and 1544 marine) and 66,209 populations (41,202 terrestrial and 25,007 marine; see Table S7 for a list of the original studies included in the analysis). Mean species richness was 67.7 and the mean duration of the time series analysed was 28 years, with the longest having 97 years. We emphasise that our study focuses only on the patterns of populations within the local assemblages included in our data, noting that we did not evaluate whether these populations would be representative of the temporal dynamics across all populations of a given species. Nonetheless, our finding of spatial variation in temporal dynamics among populations is consistent with other studies^16,27,46^, and can be particularly relevant for species with extensive distribution ranges. This is a key aspect that is clearly recognised under IUCN national and regional assessments, and which must be carefully integrated when assessing extinction risk at the global level^16,32^.

Each species in an assemblage, i.e., each population was classified into one of five distinct categories of temporal dynamics, either directional or non-directional, based on the ordered sequence of presences and absences in the assemblage time series (Fig. 1A, see ref. ^37^ for further details). To do this, we created matrices of species occurrence per year for each assemblage. First, to test whether a species was significantly increasing or decreasing through time, we split each time series evenly into an early and a late period (for time series with odd numbers of years, we assigned the midpoint to the late period). We used a contingency table analysis to check for a significant change in incidence through time, classifying each population as either increasing or decreasing. If the contingency test was not significant, there is no support for directional change. In such cases, a species may be present in every year of the time series, or assigned to the recurrent or to the random change categories, following a one-tailed runs test^79^ to check whether there were unusually small numbers of colonisations and extinctions based on the numbers of presences and absences across the entire time series. If there is support for a pattern of blocks of presences and absences, a population will be classified as recurrent (shown in pale blue in Fig. 1A), while for the random change category (darker blue shade in Fig. 1A) the pattern is not distinguishable from an equiprobable reshuffling of the observed presences and absences. We used the common significance level of *p* < 0.05 in all tests. All analyses were run in R^80^ (and see Supplementary Code file).

To quantify temporal dynamics across assemblages and test whether the temporal dynamics categories proportions differed between marine and terrestrial assemblages, we modelled the vector of proportions of the temporal categories per assemblage as a function of realm. To analyse proportional data, we first used the function DR_data() in the DirichletReg package^81^ to apply a transformation to deal with proportions equal to zero and one; specifically, y*=[y(n-1) + *1/d]/n*, where y* is the transformed proportion, *y* is the original proportion, *n* is the number of observations, and *d* is equal to five (the number of temporal categories). We then fitted a Bayesian mixed model to the transformed proportions with the Dirichlet family using the brms package^82^, having realm as a fixed effect and included a random effect nesting assemblages within studies to account for the structure of the data. The baseline category was set to random. The model fit was (Eq. 1):

1 $$\begin{array}{c}{y}^{*} \sim {Dirichlet}({\mu }_{i,j}),\\ {{logit}}({\mu }_{i,j}) \sim {\beta }_{0}{+\beta }_{1}{TemporalCategory}+{\beta }_{2}{{Realm}}_{i,j}+{\alpha }_{i,j},{\sigma }^{2}\end{array}$$

where y^*^ is the vector containing the transformed proportion values for each of the five temporal categories in assemblage *i* from study *j*, *β*_*0*_ is the global intercept (i.e. random category), *β*_*1*_ are the departures for each category, *β*_*2*_ are the departures for each category in the terrestrial realm compared to the marine realm, and *α*_*i,j*_ are the assemblage-level random effects nested with studies. The model was run with default priors and four chains, which were run for 4000 iterations (warmup = 2000; thin = 1; total post-warmup draws = 8000). The form of the model was:

2 $$	 {{\rm{bind}}} < -{{\rm{function}}}(\ldots ){{\rm{cbind}}}(\ldots ){{\rm{brm}}}\\ 	 ({{\rm{bind}}}({{\rm{Always}}}\_{{\rm{present}}},{{\rm{Random}}},{{\rm{Recurrent}}},{{\rm{Decreasing}}},{{\rm{Increasing}}}) \\ 	 \! \sim 0+{{\rm{realm}}}+(1|{{\rm{STUDY}}}\_{{\rm{ID}}}/{{\rm{assemblage}}}\_{{\rm{ID}}}),{{\rm{data}}}={{\rm{dr}}}\_{{\rm{dat}}},{{\rm{family}}} \\ 	={{\rm{dirichlet}}}({{\rm{refcat}}}={{\rm{Random}}}),{{\rm{cores}}}=2,{{\rm{chains}}}=4,{{\rm{iter}}}=4000).$$

To test whether the population temporal dynamics were systematically related to the species extinction risk, we selected the species in our data for which the IUCN Red List classification was available^10^. We excluded 15 species that were classified as Data Deficient. Thus, for this second analysis we mainly included data for vertebrates, although there were also limited matches for plants, crustaceans, and molluscs. This process resulted in 53,235 populations (40,098 terrestrial and 13,137 marine) from 973 species (551 terrestrial and 422 marine) in 943 assemblages (471 terrestrial and 472 marine) belonging to 31 original studies. We then expressed the IUCN categories as a numerical variable representing increasing extinction risk, as follows:

1= Least Concern and Lower Risk/Least Concern;

2= Near Threatened and Lower Risk/Near Threatened;

3= Vulnerable;

4= Endangered and Critically Endangered.

To ensure that our results were not overly influenced by the predominance of Least Concern species in our data, we further simplified the extinction risk categories into a binary threat classification of non-threatened vs threatened species, combining 1-2 and 3-4 in the above classification, respectively. These are two common approaches to define extinction risk responses when modelling Red List categories^11,83^. We note that while a species is classified into only one IUCN category, it can be classified into different temporal dynamics categories depending on the population’s temporal prevalence within each assemblage (Fig. 1D). We summarized the number of populations in each combination of temporal dynamics and extinction risk categories (Table S2) and implemented linear mixed models having extinction risk as a function of the temporal categories using the lmer() function in the lme4 package^84^. We also included an interaction term between temporal category and realm to test if the effects differ between marine and terrestrial species, and a random effect for assemblage to account for multiple species being sampled within each time series. The model fit was (Eq. 2):

3 $$\begin{array}{c}{y}_{i,j} \sim N\left({\mu }_{i,j},{\sigma }^{2}\right),\\ {\mu }_{i,j}={\beta }_{0}{+\beta }_{1}{TemporalCategory}+{\beta }_{2}{TemporalCategory} * {Realm}+{\alpha }_{i,j}\end{array}$$

where *y*_*i,j*_ is the extinction risk (continuous variable) of population *i* from assemblage *j*, *β*_*0*_ is the global intercept, *β*_*1*_ are the effects for each temporal category, *β*_*2*_ are the departures for each category in the terrestrial realm compared to the marine realm, and *α*_*i,j*_ are the assemblage-level random effects. The form of the model was:

4 $${{\rm{lmer}}}({{\rm{IUCN}}}\_{{\rm{risk}}} \sim \; 	 {{\rm{temporal}}}\_{{\rm{category}}} \; * \; {{\rm{realm}}} \\ 	+(1|{{\rm{assemblage}}}\_{{\rm{ID}}}),{{\rm{data}}} \\=	 \,{{\rm{all}}}\_{{\rm{data}}},{{\rm{REML}}}={{\rm{F}}})$$

We fitted two models, with either four or two extinction risk categories as described above, again having *random* as the baseline category. We calculated effect sizes using Cohen’s d, which measures the extent of difference between two group means relative to their standard deviation^85^:

5 $${Cohe}{n}^{{\prime} }{sd}=\sqrt{\frac{{t}^{2}}{{t}^{2}+{df}}}$$

where *t* is t-value and *df* degrees of freedom from the model.

Finally, both analyses were run for the entire data and for temperate regions only, where most of our data come from (Fig. S1), yielding similar results (Fig. 3 and S2, S4-S6; Tables S1 and S3–S6). We defined temperate as those studies whose locations fall wholly between the latitudes of  ± 23.5° and ± 65°, while studies with locations both inside and outside these boundaries are classified as Polar/temperate or Tropical/temperate. All non-temperate studies used here fall into one or other of these categories.

We used R packages ggeffects^86^ for plotting model results, tidyverse^87^ for data wrangling and rphylopic^88^ for the species icons used in Fig. 1.

### Reporting summary

Further information on research design is available in the Nature Portfolio Reporting Summary linked to this article.

## Supplementary information

Supplementary Infomation

Description of Additional Supplementary File

Supplementary Code 1

Reporting Summary

Transparent Peer Review file

## Data Availability

The published BioTIME data^21^ used in this study can be accessed on Zenodo (10.5281/zenodo.2602708) or through the BioTIME website (https://biotime.st-andrews.ac.uk/); links to the individual datasets are also provided in Table S7.

## Code availability

R scripts are available in Supplementary Code 1.
