# Supplementary material for: Linking species local trends from assemblage monitoring to global extinction risk
Source: Nat Commun. 2026 Jun 23;17:5071. doi: 10.1038/s41467-026-74132-7 (PMC13291242; doi:10.1038/s41467-026-74132-7)
Supplement: Supplementary file 1 — Supplementary Infomation [file 41467_2026_74132_MOESM1_ESM.pdf]

Supplementary Information for: Linking species local trends from assemblage monitoring to global extinction risk

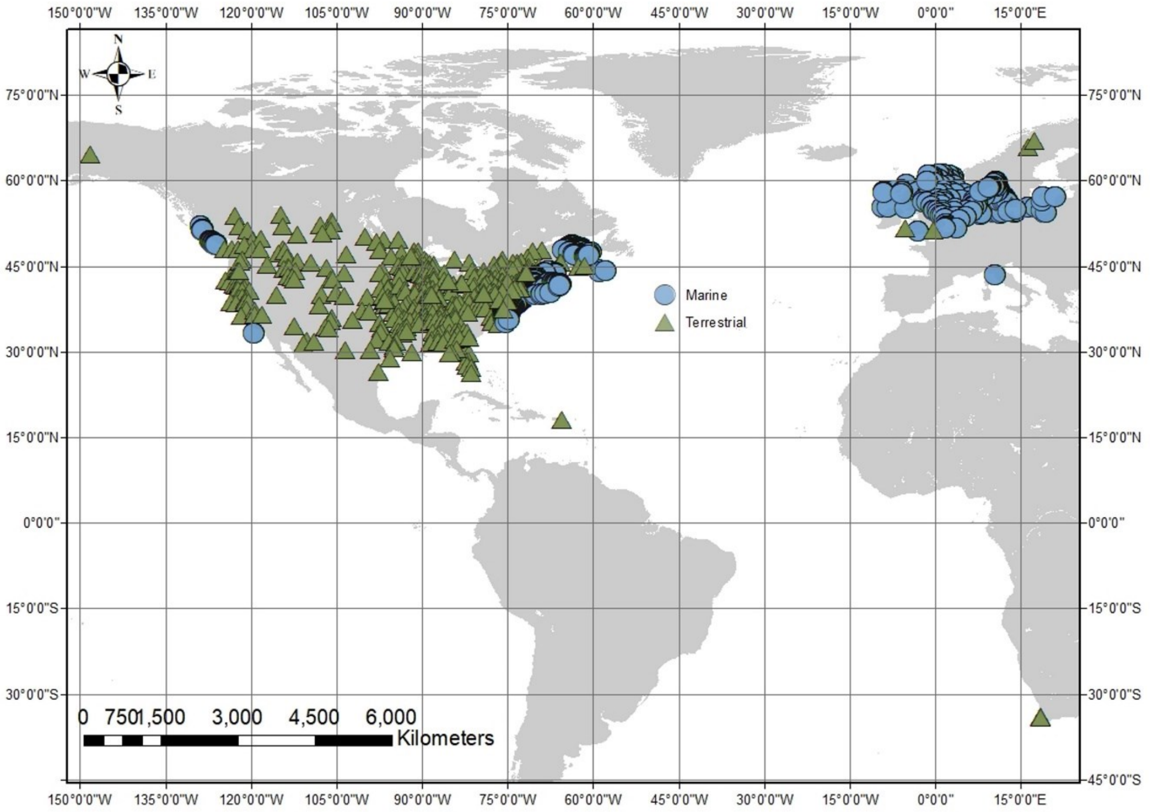

Fig. S1. Location of the central coordinates for each assemblage in our data, colored and shaped by realm, pale blue circles for Marine data and olive-green triangles for Terrestrial data. Latitude and longitude values are shown at 15° intervals around the map. These data (central coordinates) are also shown in Table S7. This map was created in ArcGIS [1].

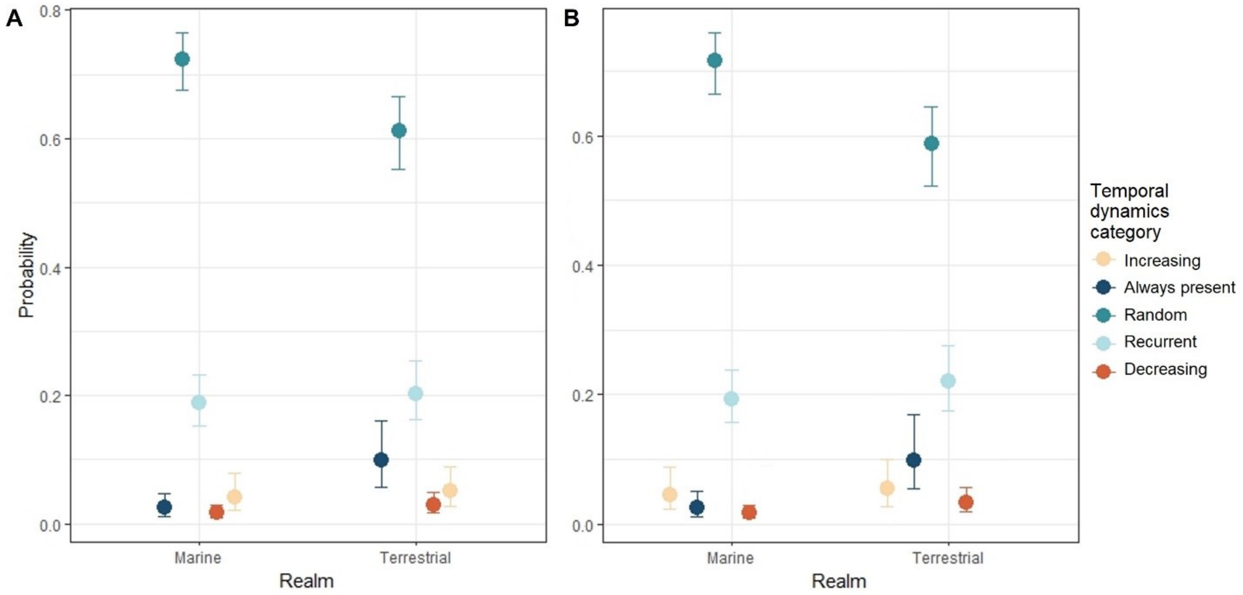

Fig. S2. Estimated proportion of populations across the temporal dynamics categories in marine and terrestrial assemblages.

The plots show the estimated values from a Bayesian mixed model with a Dirichlet distribution, where random was set as the baseline category (Table S1) with the 95% credible intervals. (A) shows the results of the model using all data whilst (B) shows the model run only with temperate assemblages (see Fig. 2 for distributions of the actual data). Proportions of distributions are as follows (total numbers of species' populations in each category in brackets): Marine: 6% (1044) Increasing, 1% (346) Decreasing, 3% (598) Always present, 74% (19335) Random, 16% (3684) Recurrent. Terrestrial: 6% (2375) Increasing, 3% (1342) Decreasing, 25% (10763) Always present, 55% (22293) Random, 11% (4429) Recurrent.

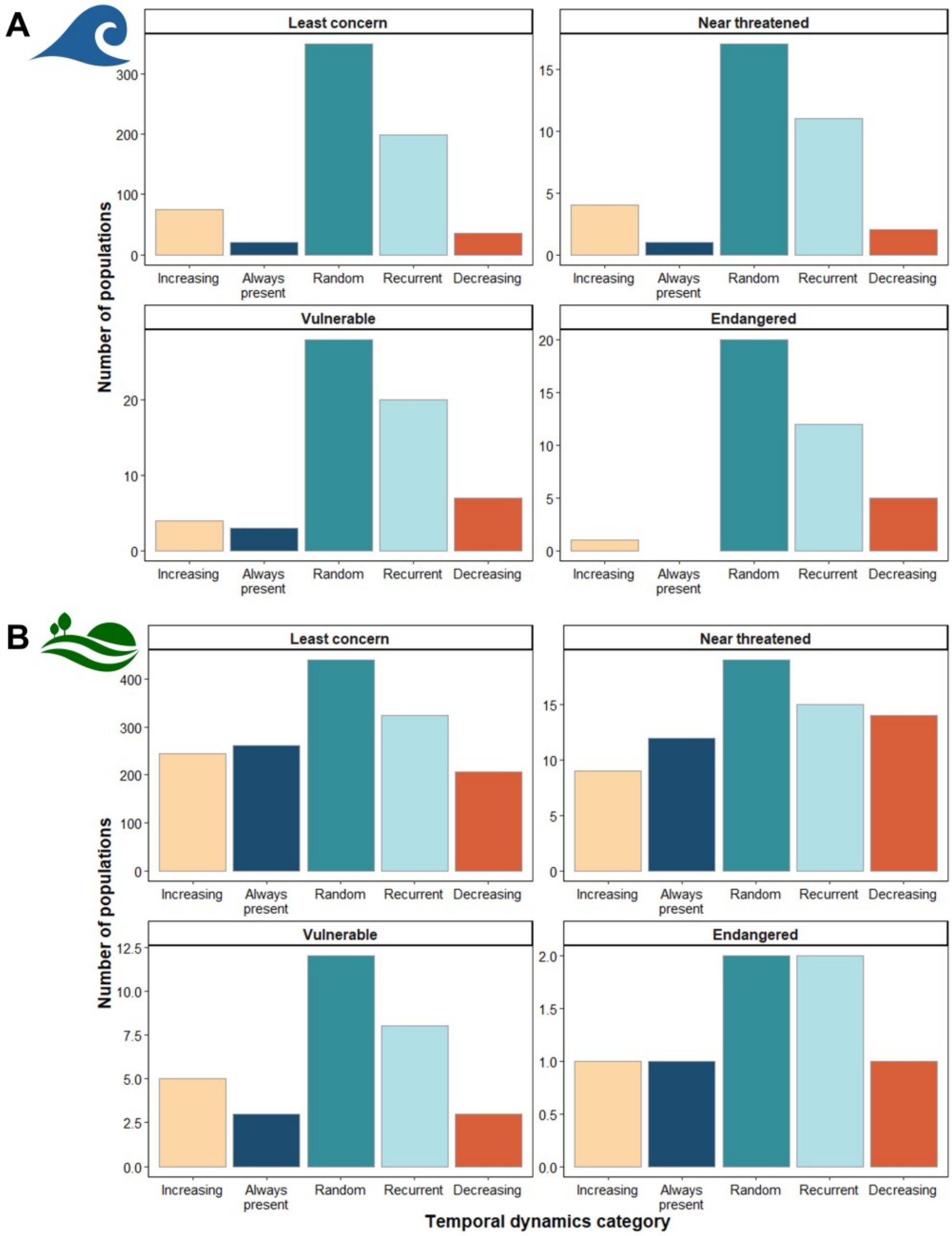

Fig. S3. Breakdown of populations by International Union for Conservation of Nature (IUCN) extinction risk and temporal dynamics category, for (A) marine and (B) terrestrial species. Note the different axes ranges; these plots correspond with the data in Fig. 3. Note that as species can be classified within different temporal dynamics categories depending upon their assemblage (Fig. 1D), the total number of populations shown here is larger than the actual species' population totals.

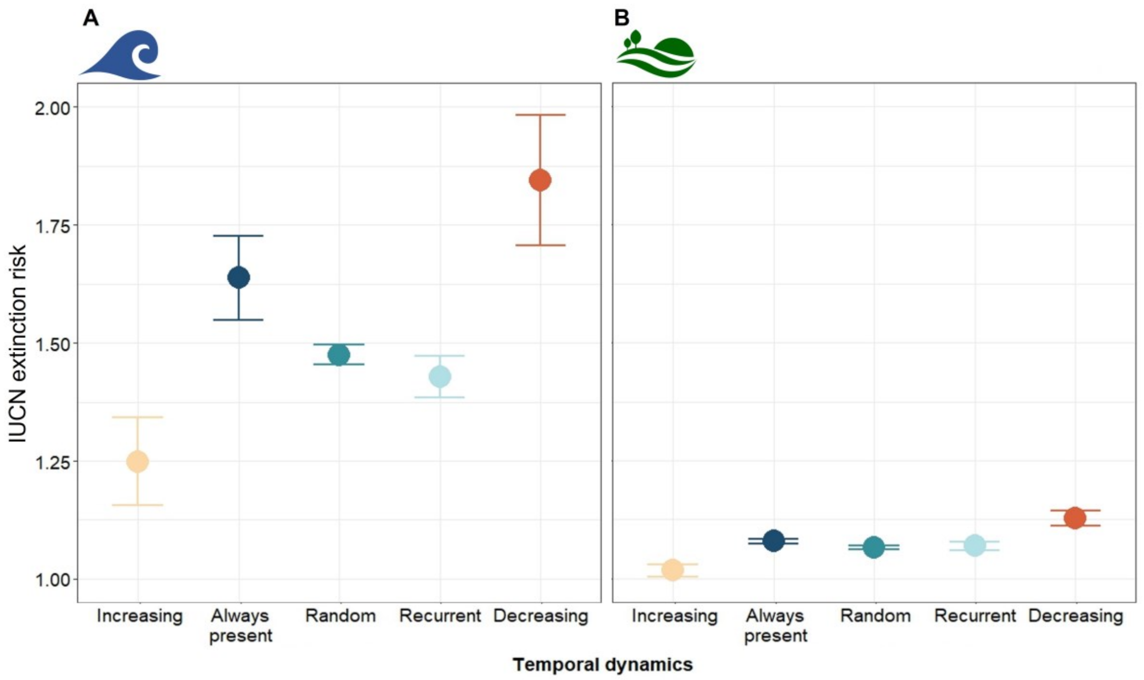

Fig. S4. Estimated relationship between global IUCN extinction risk and local population temporal dynamics within assemblages for temperate studies only, for marine (A), and terrestrial (B) species. In both plots, the error bars represent the 95% confidence intervals around the predicted values from the mixed model (two-sided model) where extinction risk was included as a continuous variable. See Figs. 3, S5 and S6 and Tables S3 – S6 for the results including all studies. Data are presented as mean values +/- 95% confidence intervals based on the standard error (SE).

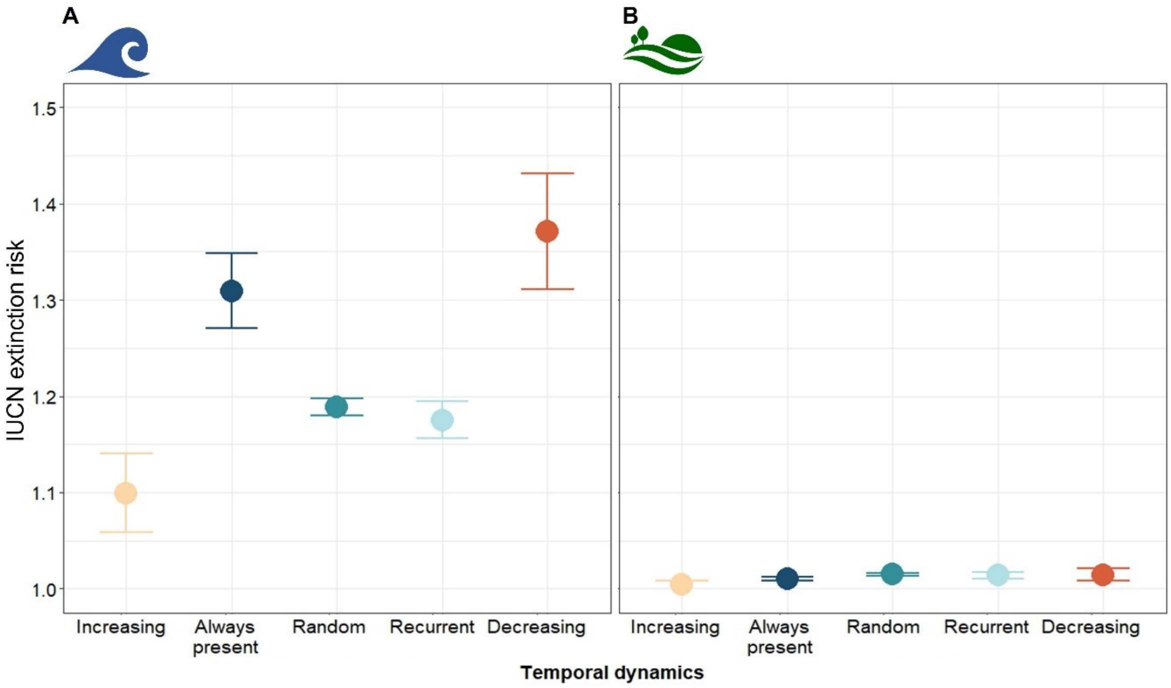

Fig. S5. Estimated relationship between global IUCN extinction risk and local population temporal dynamics within assemblages for all studies considering a binary threat classification of threatened vs non-threatened, for marine (A) and terrestrial (B) species. In both plots, the error bars represent the 95% confidence intervals around the predicted values from the mixed model (two-sided model) where extinction risk was included as a continuous variable. Data are presented as mean values +/- 95% confidence intervals based on the standard error (SE).

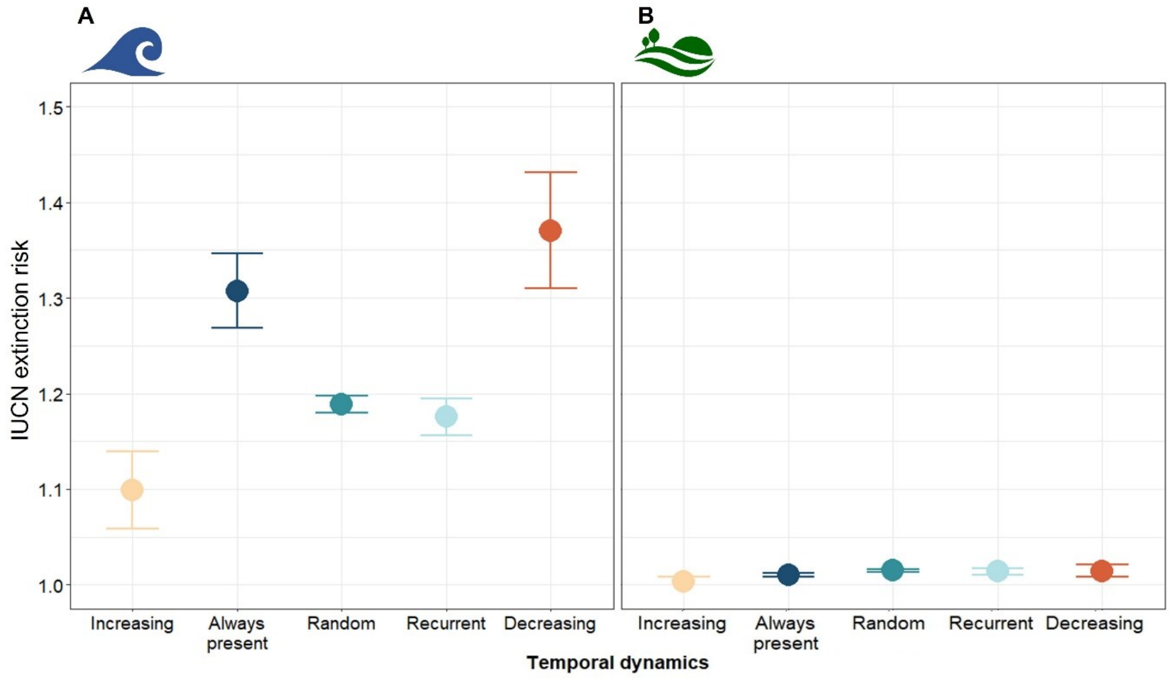

Fig. S6. Estimated relationship between global IUCN extinction risk and local population temporal dynamics within assemblages for temperate studies only and using a binary threat classification of threatened vs non-threatened, for marine (A) and terrestrial (B) species. In both plots, the error bars represent the 95% confidence intervals around the predicted values from the mixed model (two-sided model) where extinction risk was included as a continuous variable. Data are presented as mean values +/- 95% confidence intervals based on the standard error (SE).

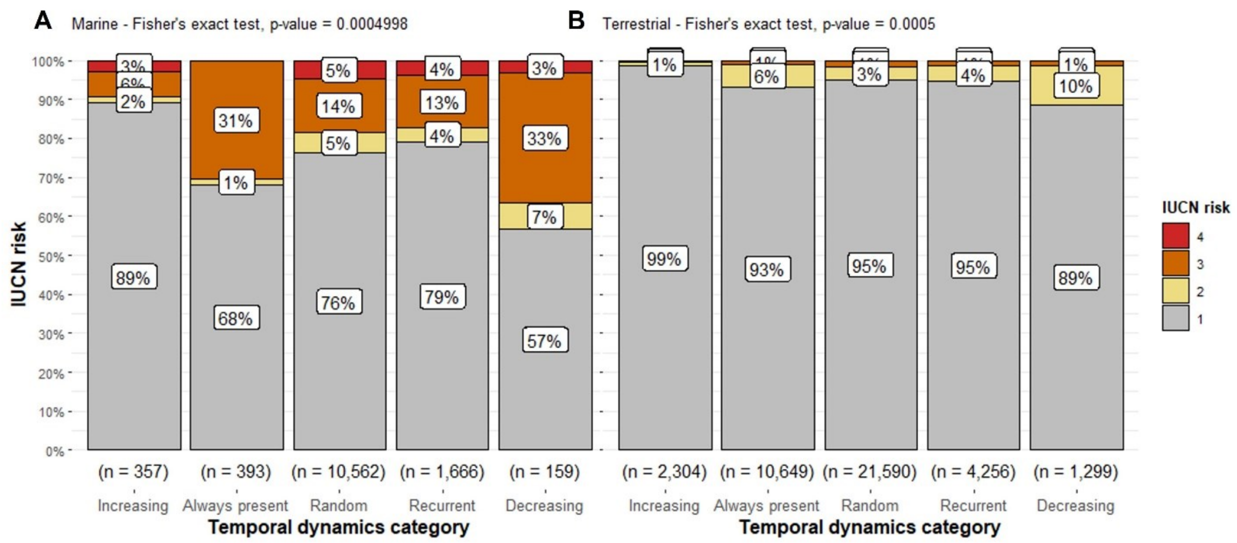

Fig. S7. Likelihood of a temporal dynamics category falling into a particular IUCN extinction risk category. The plots show the estimated percentage of a species population falling into a particular extinction risk group from each temporal dynamics category, for marine (A) and terrestrial (B) species. The results of the Fisher's exact test show that we can reject the null hypothesis and conclude that risk is influenced by temporal dynamics category.

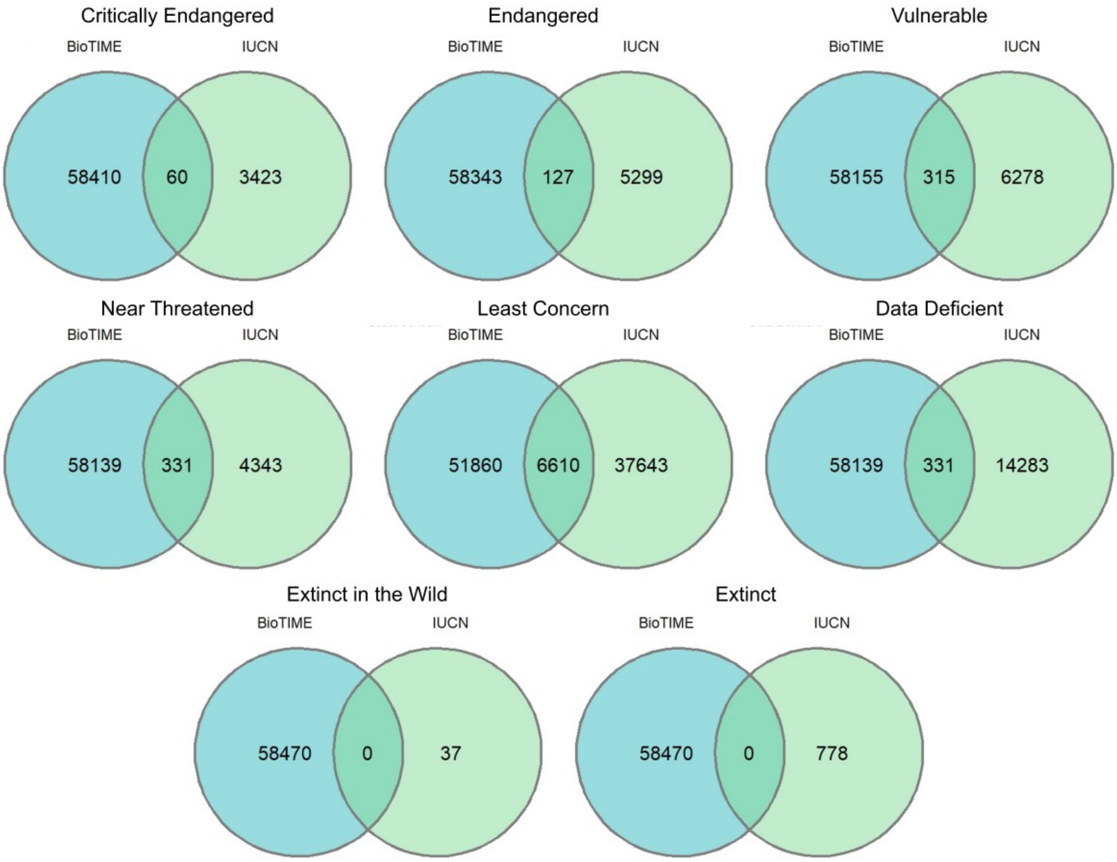

Fig. S8. Overlap of species between the BioTIME database and the IUCN Red List data. The Venn diagrams show the overlap of species in the entire BioTIME database that fall in each IUCN Red List category, illustrating that despite the comprehensive nature of both datasets, there is little overlap and therefore many species unaccounted for in either or both.

Table S1. Summaries for the models run to estimate whether the proportions across the temporal dynamics categories varied, for all assemblages (top) and for temperate assemblages only (bottom). random was set as the baseline category.

**Population-Level Effects**				
Compared to Random	**Estimate**	**Estimate error**	**lower 95% CI**	**upper 95% CI**
All studies				
Marine: Decreasing	-3.73	0.27	-4.27	-3.21
Marine: Always present	-3.38	0.35	-4.07	-2.7
Terrestrial: Decreasing	-3.01	0.25	-3.5	-2.52
Marine: Increasing	-2.85	0.35	-3.53	-2.17
Terrestrial: Increasing	-2.48	0.31	-3.11	-1.88
Terrestrial: Always present	-1.82	0.29	-2.4	-1.26
Marine: Recurrent	-1.35	0.13	-1.6	-1.08
Terrestrial: Recurrent	-1.1	0.14	-1.38	-0.82
Temperate studies only				
Marine: Decreasing	-3.76	0.29	-4.31	-3.19
Marine: Always present	-3.38	0.38	-4.12	-2.63
Terrestrial: Decreasing	-2.9	0.28	-3.45	-2.34
Marine: Increasing	-2.78	0.37	-3.51	-2.05
Terrestrial: Increasing	-2.39	0.34	-3.09	-1.71
Terrestrial: Always present	-1.79	0.33	-2.44	-1.15
Marine: Recurrent	-1.31	0.13	-1.55	-1.04
Terrestrial: Recurrent	-0.98	0.14	-1.25	-0.69

Table S2. Contingency table showing the numbers of populations within each combination of temporal dynamics and IUCN extinction risk categories.

	Marine				Terrestrial			
	Endangered	Vulnerable	Near threatened	Least concern	Endangered	Vulnerable	Near threatened	Least concern
Increasing	10	23	6	318	1	8	22	2273
Always present	0	120	5	268	1	108	621	9919
Random	494	1442	573	8053	9	315	739	20527
Recurrent	65	220	63	1318	4	56	171	4025
Decreasing	5	53	11	90	1	18	128	1152

Table S3. Summary results of the mixed model (two-sided model) fit to assess the relationship between global extinction risk and local temporal dynamics for all studies and using four categories of extinction risk; baseline is random change (see Fig. 3). σ2 refers to the within-group (or residual) variance. Marginal R^2 considers only variance of fixed effects, while conditional R^2 takes account of both fixed and random effects. Cohen's d effect sizes calculated based on $\text{Cohen's d} = \sqrt{\frac{t^2}{t^2+df}}$, where *t* is the t-value and *df* the degrees of freedom from the summary output.

**4 IUCN risk categories - all studies**					
**Predictors**	**Estimates**	**95% CI**	**p-value**	**df**	**Cohen's d**
(Intercept)	1.48	1.46 – 1.49	0.000000e+00	441.88	0.996
Always present	0.17	0.12 – 0.22	3.304740e-10	43550.13	0.030
Recurrent	-0.05	-0.08 – -0.02	3.539965e-04	51659.06	0.016
Decreasing	0.37	0.29 – 0.45	2.313322e-19	51781.47	0.040
Increasing	-0.22	-0.28 – -0.17	6.521647e-16	45941.90	0.038
Terrestrial	-0.41	-0.43 – -0.40	1.20107e-167	339.36	0.946
Terrestrial: Always present	-0.15	-0.21 – -0.10	1.800029e-08	44328.31	0.027
Terrestrial: Recurrent	0.05	0.02 – 0.08	0.001069921	52769.85	0.014
Terrestrial: Decreasing	-0.31	-0.39 – -0.22	2.622385e-12	52174.70	0.031
Terrestrial: Increasing	0.18	0.12 – 0.24	4.013378e-09	47496.09	0.027
**Random Effects**					
σ2	0.26				
N assemblage_ID	943				
Observations	53235				
Marginal R^2 / Conditional R^2	0.107 / 0.122				

Table S4. Summary results of the mixed model (two-sided model) fit to assess the relationship between global extinction risk and local temporal dynamics for temperate studies and using four categories of extinction risk; baseline is random change (see Fig. S4). σ2 refers to the within-group (or residual) variance. Marginal R^2 considers only variance of fixed effects, while conditional R^2 takes account of both fixed and random effects. Cohen's d effect sizes as in Table S3.

**4 IUCN risk categories - temperate studies only**					
**Predictors**	**Estimates**	**95% CI**	**p-value**	**df**	**Cohen's d**
(Intercept)	1.48	1.46 – 1.49	0.000000e+00	390.32	0.997
Always present	0.16	0.11 – 0.22	1.016312e-09	41257.47	0.030
Recurrent	-0.05	-0.07 – -0.02	4.984723e-04	50813.45	0.015
Decreasing	0.37	0.29 – 0.45	2.910059e-19	51086.38	0.040
Increasing	-0.23	-0.28 – -0.17	4.261765e-16	44337.59	0.039
Terrestrial	-0.41	-0.42 – -0.39	6.82284e-157	296.61	0.954
Terrestrial: Always present	-0.15	-0.20 – -0.10	4.730390e-08	42117.45	0.027
Terrestrial: Recurrent	0.05	0.02 – 0.08	0.001561589	52182.37	0.014
Terrestrial: Decreasing	-0.31	-0.39 – -0.22	2.502223e-12	51521.29	0.031
Terrestrial: Increasing	0.18	0.12 – 0.24	3.778210e-09	45977.13	0.027
**Random Effects**					
σ2	0.26				
N assemblage_ID	924				
Observations	52893				
Marginal R2 / Conditional R2	0.106 / 0.119				

Table S5. Summary results of the mixed model (two-sided model) fit to assess the relationship between global extinction risk and local temporal dynamics for all studies and using a binary threat classification of threatened vs non-threatened; baseline is random change (see Fig. S5). σ2 refers to the within-group (or residual) variance. Marginal R^2 considers only variance of fixed effects, while conditional R^2 takes account of both fixed and random effects. Cohen's d effect sizes as in Table S3.

**2 IUCN risk categories - all studies**					
**Predictors**	**Estimates**	**95% CI**	**p-value**	**df**	**Cohen's d**
(Intercept)	1.19	1.19 – 1.20	0.000000e+00	489.43	0.998
Always present	0.12	0.10 – 0.14	1.346981e-26	45472.19	0.050
Recurrent	-0.01	-0.03 – -0.00	0.01157927	52090.13	0.011
Decreasing	0.18	0.15 – 0.22	6.254954e-26	52117.99	0.046
Increasing	-0.09	-0.11– -0.07	3.424443e-14	47205.29	0.035
Terrestrial	-0.18	-0.18 – -0.17	9.82627e-176	377.99	0.938
Terrestrial: Always present	-0.12	-0.15 – -0.10	3.042692e-27	46168.38	0.050
Terrestrial: Recurrent	0.01	-0.00 – 0.03	0.04449623	52959.99	0.009
Terrestrial: Decreasing	-0.18	-0.22 – -0.15	2.632757e-23	52461.62	0.043
Terrestrial: Increasing	0.08	0.05 – 0.10	7.820784e-10	48648.40	0.028
**Random Effects**					
σ2	0.05				
N assemblage_ID	943				
Observations	53235				
Marginal R2 / Conditional R2	0.118 / 0.136				

Table S6. Summary results of the mixed model (two-sided model) fit to assess the relationship between global extinction risk and local temporal dynamics for temperate studies and using a binary threat classification of threatened vs non-threatened; baseline is random change (see Fig. S6). σ2 refers to the within-group (or residual) variance. Marginal R^2 considers only variance of fixed effects, while conditional R^2 takes account of both fixed and random effects. Cohen's d effect sizes as in Table S3.

**2 IUCN risk categories - temperate studies only**					
**Predictors**	**Estimates**	**95% CI**	**p-value**	**df**	**Cohen's d**
(Intercept)	1.19	1.19 – 1.20	0.000000e+00	442.29	0.998
Always present	0.12	0.10 – 0.14	5.779057e-26	43532.75	0.050
Recurrent	-0.01	-0.02 – -0.00	0.01809306	51372.00	0.010
Decreasing	0.18	0.15 – 0.22	6.736233e-26	51492.12	0.046
Increasing	-0.09	-0.11 – -0.07	2.933941e-14	45827.23	0.035
Terrestrial	-0.18	-0.18 – -0.17	4.40539e-167	337.48	0.946
Terrestrial: Always present	-0.12	-0.15– -0.10	1.254661e-26	44317.87	0.050
Terrestrial: Recurrent	0.01	-0.00 – 0.03	0.06621755	52456.45	0.008
Terrestrial: Decreasing	-0.18	-0.22 – -0.15	2.957748e-23	51881.96	0.044
Terrestrial: Increasing	0.08	0.05 – 0.10	9.008519e-10	47383.68	0.028
**Random Effects**					
σ2	0.05				
N assemblage_ID	924				
Observations	52893				
Marginal R2 / Conditional R2	0.117 / 0.132				

Table S7. Original studies used in the analyses, including central location, duration, taxa, climate, realm and reference number (citation).

Study ID	Realm	Climate	Taxa	Study Title	Duration	Central longitude	Central latitude	No. of species	Total records	Citation
18	Terrestrial	Temperate	Terrestrial plants	Mapped quadrats in sagebrush steppe long-term data for analyzing demographic rates and plant to plant interactions	29	44.33	-112.33	98	8034	[2]
39	Terrestrial	Temperate	Birds	Bird community dynamics in a temperate deciduous forest Long-term trends at Hubbard Brook	45	43.91	-71.75	52	959	[3-6]
42*	Terrestrial	Temperate	Birds	Eastern Wood	30	51.2965	-0.38352	45	954	[7-13]
46	Terrestrial	Temperate	Birds	Skokholm Bird Observatory	47	51.698	-5.277	29	528	[14]
47	Terrestrial	Temperate	Birds	Detection of Density-Dependent Effects in Annual Duck Censuses	26	50.84545	-107.446	13	392	[15]
54	Terrestrial	Tropical	Terrestrial invertebrates	El Verde Grid invertebrate data (Big Grid Snail Captures 1991-2007)	24	18.1667	-65.5	19	21702	[16]
56	Terrestrial	Temperate	Mammals	Small Mammal Mark-Recapture Population Dynamics at Core Research Sites	20	34.2	-106.43	28	16657	[17]
59	Terrestrial	Temperate	Mammals	Long-term monitoring and experimental manipulation of a Chihuahuan Desert ecosystem near Portal. Arizona. USA	26	30.3226	-103.501	29	427	[18]
67	Terrestrial	Temperate	Birds	Animal Demography Unit - Coordinated Waterbird Counts (CWAC) (AfrOBIS)	24	-28.95447	24.95096	68	15448	[19]

78	Marine	Temperate	Benthos	IOW Macrozoobenthos monitoring Baltic Sea (1980-2005) (EurOBIS)	25	56.72964	18.23636	212	3587	[20]
100*	Marine	Temperate	Fish	Community level response to climate change, long-term study of the fish community of the Bristol Channel	31	51.14	-3.08	83	5199	[21,22]
101*	Marine	Temperate	Marine invertebrates	Community level response to climate change, long-term study of the fish and crustacean community of the Bristol Channel	31	51.14	-3.08	15	2210	[21,22]
119	Marine	Temperate	Fish	DFO Maritimes Research Vessel Trawl Surveys Fish Observations (OBIS Canada)	41	43.98743	-63.6697	231	121804	[23]
178	Marine	Temperate	Fish	Pacific Shrimp Trawl Survey (OBIS Canada)	39	53.54327	-140.266	476	128311	[24]
180	Marine	Polar/ Temperate	Fish	ECNASAP - East Coast North America Strategic Assessment (OBIS Canada)	26	37.77056	-50.7927	273	410802	[25]
182	Marine	Temperate	All	Snow crab research trawl survey database (Southern Gulf of St. Lawrence. Gulf region. Canada) from 1988 to 2010 (OBIS Canada)	22	47.48092	-62.7617	33	35005	[26]
195	Terrestrial	Temperate	Birds	Breeding birds survey North America	30	40.80924	-96.1873	385	699449	[27]
196	Marine	Temperate	Benthos	SOTEAG Rocky Shore Survey (Sullom Voe)	35	60.46645	-1.32288	252	91491	[28]
197*	Marine	Temperate	Fish	Scottish West Coast Surveys - all species of fish (1985 - 2013)	28	56.52116	-6.52895	149	279726	[29]
198*	Marine	Temperate	Fish	Baltic international demersal trawl surveys	23	56.3595	16.42173	142	751021	[30]

200	Marine	Temperate	Marine invertebrates	NEFSC Benthic Database (OBIS-USA)	30	35.69171	-74.0908	2105	102143	[31]
210*	Marine	Temperate	Fish	ICES North Sea International Bottom Trawl Survey for commercial fish species. ICES Database of trawl surveys (DATRAS)	47	56.46378	3.500367	254	296524	[32]
213	Marine	Temperate	All	Northeast Fisheries Science Center Bottom Trawl Survey Data (OBIS-USA)	48	36.62513	-72.636	1023	439452	[33]
214	Terrestrial	Temperate	Terrestrial plants	Long-term growth mortality and regeneration of trees in permanent vegetation plots in the Pacific Northwest 1910 to present	88	45.34296	-122.799	39	37350	[34]
215*	Terrestrial	Temperate/Tropical	Birds	Hawk Migration Association of North America (HMANA)	57	38.40865	-99.5157	39	991769	[35]
221	Terrestrial	Temperate	Terrestrial plants	Vegetation Plots of the Bonanza Creek LTER Control Plots Species Count (1975 - 2004)	26	64.84423	-148.052	52	1157	[36]
243	Terrestrial	Temperate	Terrestrial plants	Long-term N-fertilized vegetation plots on Hog Island Virginia Coastal Barrier Islands 1992 to 2014	22	37.44663	-75.6675	51	8508	[37,38]
300	Terrestrial	Temperate	Terrestrial invertebrates	Insect Populations via Sticky Traps at KBS-LTER (Kellogg Biological Station. MI)	25	42.40885	-85.3832	21	47798	[39]
308	Terrestrial	Temperate	Mammals	Powdermill Nature Reserve monitored small mammal populations from 1979-1999.	21	40.17074	-79.2602	14	35398	[40]

311	Terrestrial	Temperate	Mammals	Seasonal summary of numbers of small mammals on 14 LTER traplines in prairie habitats at Konza Prairie	33	39.08333	-96.5833	15	2458	[41]
333	Terrestrial	Temperate	Birds	Weekly record of bird species observed on Konza Prairie	29	39.08333	-96.5833	132	9261	[42]
339	Terrestrial	Temperate	Birds	Species trends turnover and composition of a woodland bird community in southern Sweden during a period of 57 years.	57	55.71667	13.33333	39	1210	[43]
363	Terrestrial	Temperate	Birds	The 37-year dynamics of a subalpine bird community with special emphasis on the influence of environmental temperature and Epirrita autumnata cycles.	37	65.96806	16.31666	35	636	[44]
366	Terrestrial	Temperate	Mammals	Small Mammal Exclosure Study (SMES)	25	34.35	-106.88	24	3389	[45]
379	Marine	Temperate	Marine invertebrates	Calafuria Low-shore Intertidal Dataset (1991-2014)	22	43.46937	10.33596	61	1153	[46]
413	Terrestrial	Temperate	Birds	Bird populations in east central Illinois. Fluctuations variations and development over a half-century	44	39.98333	-88.65	60	1196	[47]
414	Terrestrial	Temperate	Birds	Bird populations in east central Illinois. Fluctuations variations and development over a half-century	48	39.98333	-88.65	48	963	[47]
416	Terrestrial	Temperate	Birds	Bird populations in east central Illinois. Fluctuations variations and development over a half-century	25	40.13333	-88.3	53	766	[47]

420	Terrestrial	Polar/ Temperate	Birds	Species composition and population fluctuations of alpine bird communities during 38 years in the Scandinavian mountain range	38	67.077	17.435	47	1010	[48]
428	Marine	Temperate	All	Long term monitoring of fish abundances from coastal Skagerrak	97	58.95856	9.768152	59	101221	[49-52]
466	Marine	Temperate	Fish	Trawl Survey Data from Rockall Scotland (1986 - 2008)	23	56.99054	-9.07255	94	27592	[53]
468	Marine	Temperate	Benthos	A multi-decade time series of kelp forest community structure at San Nicolas Island California.	31	33.24543	-119.509	151	27700	[54]

*study not publicly available in BioTIME

References

1. ESRI. ArcGIS Desktop: Release 10.7, Redlands, CA: Environmental Systems Research Institute. https://www.esri.com (2023)
2. Zachmann, L., Moffet, C. & Adler, P. Mapped quadrats in sagebrush steppe: long-term data for analyzing demographic rates and plant–plant interactions. *Ecology* **91**, 3427-3427 (2010).
3. Holmes, R. & Sturges, F. Bird community dynamics and energetics in a northern hardwoods ecosystem. *The Journal of Animal Ecology* **1**, 175-200 (1975).
4. Holmes, R. T. & Sherry, T. W. Assessing population trends of New Hampshire forest birds: local vs. regional patterns. *The Auk* **105**, 756-768 (1988).
5. Holmes, R. T. & Sherry, T. W. Thirty-year bird population trends in an unfragmented temperate deciduous forest: importance of habitat change. *The Auk* **118**, 589-609 (2001).
6. Holmes, R. T., Sherry, T. W. & Sturges, F. W. Bird Community Dynamics in a Temperate Deciduous Forest: Long-Term Trends at Hubbard Brook. *Ecological Monographs* **56**, 201-220 (1986).
7. Gibbons, D., Reid, J. & Chapman, R. The new atlas of breeding birds in Britain and Ireland: 1988-1991. *London: T. & AD Poyser* (1988).
8. Lack, P. *The atlas of wintering birds in Britain and Ireland*. (A&C Black, 2010).
9. Stone, B. *et al.* Population estimates of birds in Britain and in the United Kingdom. *British Birds* **90**, 1-22 (1997).
10. Beven, G. Changes in breeding bird populations of an oak-wood on Bookham Common, Surrey, over twenty-seven years. *London Naturalist* **55**, 23-42 (1976).
11. Williamson, M. in *Symposium of the British Ecological Society.*
12. Standley, P., Bucknell, N., Swash, A. & Collins, I. *The Birds of Berkshire*. (Berkshire Atlas Group, 1996).
13. Gaston, K. J. & Blackburn, T. M. *Pattern and process in macroecology.*, (Wiley-Blackwell, 2000).
14. Williamson, M. The land-bird community of Skokholm: ordination and turnover. *Oikos*, 378-384 (1983).
15. Vickery, W. L. & Nudds, T. D. Detection of Density-Dependent Effects in Annual Duck Censuses. *Ecology* **65**, 96-104 (1984).
16. Willig, M. R. El Verde long-term invertebrate data - Luquillo forest dynamics plot (LFDP) ver 9996737 (Version 9996737). Environmental Data Initiative. Available at https://doi.org/10.6073/pasta/45e3a90ed462f66acdde83636746f87f, accessed 2016. (2016).
17. Friggens, M. SEV008 - Sevilleta LTER Small Mammal Population data. Sevilleta LTER. Available at: https://sevlter.unm.edu/data/sev-008/4786, accessed 2012. (2008).
18. Ernest, S., Valone, T. J. & Brown, J. H. Long-term monitoring and experimental manipulation of a Chihuahuan Desert ecosystem near Portal, Arizona, USA. *Ecology* **90**, 1708-1708 (2009).
19. Animal Demography Unit. Coordinated waterbird counts (CWAC). AfrOBIS. Available at: https://obis.org/dataset/a60f84ee-0e25-428e-b171-16686b554702, accessed 2012. (2012).
20. Zettler, M. L. Macrozoobenthos Baltic Sea (1980-2005) as part of the IOW-monitoring. Institut für Ostseeforschung Warnemünde, Germany. Available at: https://ipt.vliz.be/eurobis/resource?r=macroben_zettler_evco, accessed 2012. (2005).

21 Henderson, P. A. & Magurran, A. E. Data from: Direct evidence that density-dependent regulation underpins the temporal stability of abundant species in a diverse animal community. Available at Dryad Data Repository. http://dx.doi.org/10.5061/dryad.3090c, accessed 2014. doi:http://dx.doi.org/10.5061/dryad.3090c (2014).
22 Henderson, P. A. The long-term study of the fish and crustacean community of the Bristol Channel. Available at http://www.pisces-conservation.com/, accessed 2013.
23 Clark, D. & Branton, B. DFO Maritimes Research Vessel Trawl Surveys, OBIS Canada Digital Collections. Bedford Institute of Oceanography, Dartmouth, Nova Scotia, Canada, OBIS Canada. Available at: https://www.gbif.org/dataset/86711916-f762-11e1-a439-00145eb45e9a, accessed 2012. (2007).
24 Boutillier, J. A. Pacific shrimp trawl survey. Fisheries; Oceans Canada.Bedford Institute of Oceanography; OBIS Canada Digital Collections. OBIS SEAMAP. Available at: http://seamap.env.duke.edu/dataset/103150261, accessed 2012. (2007).
25 Brown, S. K. R., Zwanenburg, K. & Branton, R. ECNASAP East Coast of North America groundfish data. OBIS. Available at: https://obis.org/dataset/11bc2cb2-a837-4cd1-9450-52e3bb427cf6, accessed 2012. (2005).
26 Wade, E. J. Snow crab research trawl survey database (Southern Gulf of St. Lawrence, Gulf region, Canada) from 1988 to 2010. OBIS Canada, Bedford Institute of Oceanography, Dartmouth, Nova Scotia, Canada. Available at: https://obis.org/dataset/b2fe1445-9bb8-4849-962b-0696d0e3e29f, accessed 2012. (2011).
27 Pardieck, K. L., Ziolkowski Jr., D. J. & Hudson, M.-A. R. North American breeding bird survey dataset 1966 - 2014, version 2014.0 (Version 2014). U.S. Geological Survey, Patuxent Wildlife Research Center. Available at: www.pwrc.usgs.gov/BBS/RawData/, accessed 2014. (2014).
28 Moore, J. J. & Howson, C. M. Survey of the rocky shores in the region of Sullom Voe, Shetland, July 2013 (pp. 30 pp + iv). A report to SOTEAG from Aquatic Survey & Monitoring Ltd., Cosheston, Pembrokeshire. Available at: https://www.soteag.org.uk/environmental-monitoring/monitoring-reports/, accessed 2013. (2013).
29 DATRAS. ICES Scottish West Coast Bottom Trawl Survey (SWC-IBTS) 1985-2014. Available at https://datras.ices.dk, accessed 2015. (2015).
30 DATRAS. ICES Baltic International Trawl Survey For Commercial Fish Species (1991 - 2013). Available at https://datras.ices.dk, accessed 2013. (2013).
31 NEFSC. Northeast Fisheries Science Center, NOAA National Marine Fisheries Service, & U.S. Department of Commerce. (2010). NEFSC benthic database. Northeast Fisheries Science Center, 166 Water Street, Woods Hole Laboratories, Woods Hole, MA 02543. Available at: http://www.usgs.gov/obis-usa/, accessed 2012. (2010).
32 DATRAS. ICES North Sea International Bottom Trawl Survey For Commercial Fish Species 1965-2019. Available at https://datras.ices.dk, accessed 2019. (2019).
33 NMFS. NOAA’s National Marine Fisheries Service. Northeast Fisheries Science Center. Northeast fisheries science center bottom trawl survey data NOAA’s National Marine Fisheries Service (NMFS) Northeast Fisheries Science Center, Ecosystems Survey Branch. Woods Hole, Massachusetts, United States of America. Available at: https://www1.usgs.gov/obis-usa/ipt/resource?r=nefsc_bottom_trawl_surveys_coml, accessed 2013. (2005).
34 Harmon, M. & Franklin, J. Long-term growth, mortality and regeneration of trees in permanent vegetation plots in the Pacific Northwest, 1910 to present. Forest Science

Data Bank; Environmental Data Initiative. Available at: https://doi.org/10.6073/pasta/2315afa15ad0a2317b49565da6258c47, accessed 2012. (2012).
35 HMANA. Hawk Migration Association of North America (HMANA). Available at: http://www.hmana.org/, accessed 2012. (2012).
36 Viereck, L. A., Van Cleve, K., Chapin, F. S., Ruess, R. W. & Hollingsworth, T. N. Vegetation plots of the Bonanza Creek LTER control plots: Species count (1975 - 2004) ver 20 (Version 20). Environmental Data Initiative. Available at: https://doi.org/10.6073/pasta/8dd0e1ac48e2f82b51adabfbd3c62ae2, accessed 2012. doi:http://dx.doi.org/10.6073/pasta/8dd0e1ac48e2f82b51adabfbd3c62ae2. (2005).
37 Day, F. P. Long-term N-fertilized vegetation plots on Hog Island, Virginia Coastal Barrier Islands, 1992-2014. Environmental Data Initiative. Available at: https://doi.org/10.6073/pasta/206d661aaaf992eba3c7714fd683331a, accessed 2014. (2014).
38 Day, F. P., Conn, C., Crawford, E. & Stevenson, M. Long-term effects of nitrogen fertilization on plant community structure on a coastal barrier island dune chronosequence. *Journal of Coastal Research*, 722-730 (2004).
39 Landis, D. & Gage, S. Insect population dynamics on the main cropping system experiment at the Kellogg Biological Station, Hickory Corners, MI (1989 to 2014). Environmental Data Initiative. Available at: https://doi.org/10.6073/pasta/f0776c1574808b08c484c1f7645a7357, accessed 2016. (2014).
40 Merritt, J. Long term mammal data from powdermill biological station 1979-1999 (Version 21) Environmental Data Initiative. Available at: https://doi.org/10.6073/pasta/086fda03bd91ce9c2331e3a6fdd9bcd1, accessed 2016. doi:http://dx.doi.org/10.6073/pasta/83c888854e239a79597999895bb61cfe (1999).
41 Kaufman, D. W. CSM01 Seasonal Summary of Numbers of Small Mammals on 14 LTER Traplines in Prairie Habitats at Konza Prairie ver 8. Environmental Data Initiative. Available at: https://doi.org/10.6073/pasta/9735a16a0018d85ff5efb8b74fd100f4, accessed 2016 (2016).
42 Sandercock, B. K. CBP01 variable distance line-transect sampling of bird population numbers in different habitats on konza prairie (Version 8). Environmental Data Initiative. Available at: https://doi.org/10.6073/pasta/7a4ad4fda7ce4b0696ee959fe1686d72, accessed 2016. (2016).
43 Svensson, S., Thorner, A. & Nyholm, N. Species trends, turnover and composition of a woodland bird community in southern Sweden during a period of fifty-seven years. *Ornis Svecica* **20**, 31-44 (2010).
44 Kartzinel, T. R. *et al.* Plant and small-mammal responses to large-herbivore exclusion in an African savanna: five years of the UHURU experiment. *Ecology* **95**, 787-787 (2014).
45 Lightfoot, D. Small Mammal Exclosure Study (SMES) Leaf Litter Study in the Chihuahuan Desert Grassland and Shrubland at the Sevilleta National Wildlife Refuge, New Mexico ver 128607. Environmental Data Initiative. Available at: https://doi.org/10.6073/pasta/2f99420037c88c1c751b38ea8f3a91b1, accessed 2016. (2016).
46 Benedetti-Cecchi, L. "Calafuria Low-shore Intertidal Dataset (1991-2014)". Department of Biology, University of Pisa. Accessed 2016.

47 Kendeigh, S. C. Bird populations in east central Illinois: Fluctuations, variations, and development over a half-century. *University of Illinois Press.* (1982).
48 Svensson, S. Species composition and population fluctuations of alpine bird communities during 38 years in the Scandinavian mountain range. *Ornis Svecica* **16**, 183-210 (2006).
49 Barceló, C., Ciannelli, L., Olsen, E. M., Johannessen, T. & Knutsen, H. Eight decades of sampling reveal a contemporary novel fish assemblage in coastal nursery habitats. *Global Change Biology* **22**, 1155-1167 (2016).
50 Olsen, E. M., Carlson, S. M., Gjøsæter, J. & Stenseth, N. C. Nine decades of decreasing phenotypic variability in Atlantic cod. *Ecology Letters* **12**, 622-631 (2009).
51 Rogers, L. A. *et al.* Climate and population density drive changes in cod body size throughout a century on the Norwegian coast. *Proceedings of the National Academy of Sciences* **108**, 1961-1966, doi:10.1073/pnas.1010314108 (2011).
52 Stenseth, N. C. *et al.* Dynamics of coastal cod populations: intra- and intercohort density dependence and stochastic processes. *Proceedings of the Royal Society of London. Series B: Biological Sciences* **266**, 1645-1654, doi:10.1098/rspb.1999.0827 (1999).
53 Neat, F. & Campbell, N. Demersal fish diversity of the isolated Rockall plateau compared with the adjacent west coast shelf of Scotland. *Biological Journal of the Linnean Society* **104**, 138-147 (2011).
54 Kenner, M. C. *et al.* A multi-decade time series of kelp forest community structure at San Nicolas Island, California (USA). *Ecology* **94**, 2654-2654, doi:10.1890/13-0561R.1 (2013).
