# Supplementary material for: Linking species local trends from assemblage monitoring to global extinction risk
Source: Nat Commun. 2026 Jun 23;17:5071. doi: 10.1038/s41467-026-74132-7 (PMC13291242; doi:10.1038/s41467-026-74132-7)
Supplement: Supplementary file 2 — Description of Additional Supplementary File [file 41467_2026_74132_MOESM2_ESM.pdf]

Description of Additional Supplementary File

Supplementary Code 1 -

The code shown provides methods and models used in our manuscript, including data handling, statistical models and assignation of temporal dynamics categories. Each section has a description of the succeeding code block and any instructions on use required.
