# Supplementary material for: Linking species local trends from assemblage monitoring to global extinction risk
Source: Nat Commun. 2026 Jun 23;17:5071. doi: 10.1038/s41467-026-74132-7 (PMC13291242; doi:10.1038/s41467-026-74132-7)
Supplement: Supplementary file 3 — Supplementary Code 1 [file 41467_2026_74132_MOESM3_ESM.pdf]

Code for: Linking species local trends from assemblage monitoring to global extinction risk

Load libraries

```
library(DirichletReg)
library(tidyverse)
library(tseries)
library(dggridR)
library(brms)
library(lme4)
```

Read in downloaded BioTIME query and subset by selected studies, these are filtered by realm and length of time series. Note that 16% of the studies used in these analyses are not held in the BioTIME database, they are publicly available elsewhere and can be sourced using the relevant links below.

Study ID	Source or link
42	Appendix II of Pattern and process in macroecology. (Gaston & Blackburn)
100 & 101	Dryad repository - http://dx.doi.org/10.5061/dryad.3090c
197	SWC-IBTS dataset from - https://datras.ices.dk
198	BITS dataset from - https://datras.ices.dk
210	NS-IBTS dataset from - https://datras.ices.dk
215	Hawk count downloads from - http://www.hmana.org/

biotime = downloaded query

```
studies<-c(18,39,46,47,54,56,59,67,78,119,178,180,182,195,196,200,
213,214,221,243,300,308,311,333,339,363,366,379,413,414,416,420,428,466,468)

subBio<-subset(biotime, STUDY_ID %in% studies)
```

Gridding function used to account for the heterogeneity in spatial extent across the different studies and quantify temporal patterns using a common spatial resolution across studies and realms.

Parameters btf = subset biotime query (subBio) meta = downloaded meta data from the BioTIME site

```r
gridding <- function(meta, btf) {
  bt <- dplyr::inner_join(meta, btf, by = 'STUDY_ID') %>%
    dplyr::rename(Species = valid_name)

  meta <- meta %>%
    dplyr::mutate(StudyMethod = dplyr::if_else(NUMBER_LAT_LONG == 1, "SL", NA))
  bt <- bt %>%
    dplyr::mutate(StudyMethod = dplyr::if_else(NUMBER_LAT_LONG == 1, 'SL', 'ML'))

  SL_extent_mean <- meta %>%
    dplyr::filter(StudyMethod == 'SL' & AREA_SQ_KM <= 500) %>%
    dplyr::summarise(extent_mean = mean(AREA_SQ_KM, na.rm = TRUE)) %>%
    dplyr::pull(extent_mean)
  SL_extent_sd <- meta %>%
    dplyr::filter(StudyMethod == 'SL' & AREA_SQ_KM <= 500) %>%
    dplyr::summarise(extent_sd = stats::sd(AREA_SQ_KM, na.rm = TRUE)) %>%
    dplyr::pull(extent_sd)

  bt <- bt %>% dplyr::mutate(
    StudyMethod = dplyr::if_else(
      condition = AREA_SQ_KM < (SL_extent_mean + SL_extent_sd),
      true = 'SL',
      false = StudyMethod)
  )

  bt <- bt %>%
    dplyr::mutate(
      lon_to_grid = dplyr::if_else(StudyMethod == 'SL', CENT_LONG, LONGITUDE),
      lat_to_grid = dplyr::if_else(StudyMethod == 'SL', CENT_LAT, LATITUDE))

  oneyear <- bt %>%
    dplyr::group_by(STUDY_ID) %>%
    dplyr::filter(max(YEAR) - min(YEAR) == 0) %>%
    dplyr::summarise() %>%
    dplyr::collect() %>%
    dplyr::pull(STUDY_ID)

  bt <- bt %>% dplyr::filter(!(STUDY_ID %in% oneyear))

  dgg <- dggridR::dgconstruct(res = 12)
  res <- dggridR::dg_closest_res_to_area(dgg, SL_extent_mean + SL_extent_sd)
  dgg <- dggridR::dgsetres(dgg, res)
  bt <- as.data.frame(bt)

  bt$cell <- dggridR::dgGEO_to_SEQNUM(dgg, bt$lon_to_grid, bt$lat_to_grid)$seqnum

  check <- bt %>%
    dplyr::group_by(StudyMethod, STUDY_ID) %>%
    dplyr::summarise(n_cell = dplyr::n_distinct(cell))

  if (sum(dplyr::filter(check, StudyMethod == 'SL') %>% .$n_cell != 1) == 0) {
    print("all SL studies have 1 grid cell")
  } else { print("ERROR: some SL studies have > 1 grid cell") }

  check2 <- bt %>%
    dplyr::group_by(StudyMethod, STUDY_ID, YEAR) %>%
```

```r
  dplyr::summarise(n_cell = dplyr::n_distinct(cell))

# range(check2$n_cell)

bt <- bt %>%
  tidyr::unite(col = rarefyID, STUDY_ID, cell, sep = "_", remove = FALSE)

rarefyID_coords_nest <- bt %>%
  dplyr::ungroup() %>%
  dplyr::filter(StudyMethod != 'SL') %>%
  dplyr::select(STUDY_ID, rarefyID, LONGITUDE, LATITUDE) %>%
  dplyr::distinct(rarefyID, LONGITUDE, LATITUDE, .keep_all = TRUE) %>%
  dplyr::group_by(rarefyID) %>%
  dplyr::mutate(n_locations = dplyr::n_distinct(LONGITUDE,LATITUDE)) %>%
  dplyr::ungroup() %>%
  dplyr::filter(n_locations > 1) %>%
  dplyr::select(-n_locations) %>%
  dplyr::group_by(STUDY_ID, rarefyID) %>%
  tidyr::nest()

cell_extent <- numeric()
centre_rarefyID_x <- numeric()
centre_rarefyID_y <- numeric()
vertices_check <- data.frame()

for (i in 1:nrow(rarefyID_coords_nest)) {
  ## check what it is doing
  print(paste('rarefyID', i, 'of',
              length(unique(rarefyID_coords_nest$rarefyID))))
  hull <- grDevices::chull(
    x = unlist(rarefyID_coords_nest$data[[i]][,'LONGITUDE']),
    y = unlist(rarefyID_coords_nest$data[[i]][,'LATITUDE']))

  vertices <- rarefyID_coords_nest$data[[i]][hull, c('LONGITUDE', 'LATITUDE')]
  info <- cbind.data.frame(
    Realm = rep(x = rarefyID_coords_nest$STUDY_ID[i],
                times = nrow(vertices)),
    rarefyID = rep(x = rarefyID_coords_nest$rarefyID[i],
                   times = nrow(vertices)),
    vertices)
  vertices_check <- rbind.data.frame(vertices_check, info)

  ## get the extent of cells in km2
  cell_extent[i] = geosphere::areaPolygon(data.frame(
    x = vertices$LONGITUDE,
    y = vertices$LATITUDE))
  ## get the centre points of the cells
  centre_rarefyID_x[i] = geosphere::geomean(
    cbind(x = vertices$LONGITUDE,
          y = vertices$LATITUDE))[1]
  centre_rarefyID_y[i] = geosphere::geomean(
    cbind(x = vertices$LONGITUDE,
          y = vertices$LATITUDE))[2]
}

rarefyID_cell_centre <- cbind.data.frame(
  rarefyID_coords_nest[, 1:2],
  cell_extent,
```

```
    rarefyID_x = centre_rarefyID_x,
    rarefyID_y = centre_rarefyID_y)
  rarefyID_cell_centre <- dplyr::as_tibble(rarefyID_cell_centre)

  SL_coords <- bt %>%
    dplyr::ungroup() %>%
    dplyr::filter(StudyMethod == 'SL') %>%
    dplyr::select(STUDY_ID, rarefyID, CENT_LONG, CENT_LAT) %>%
    ## cell extent = 0, need to rename the centre points to merge
    dplyr::mutate(cell_extent = 0,
                  rarefyID_x = CENT_LONG,
                  rarefyID_y = CENT_LAT) %>%
    dplyr::select(-CENT_LONG, -CENT_LAT)

  ML_coords <- dplyr::ungroup(bt) %>%
    dplyr::filter(StudyMethod != 'SL') %>%
    dplyr::select(STUDY_ID, rarefyID, LONGITUDE, LATITUDE) %>%
    dplyr::distinct(rarefyID, LONGITUDE, LATITUDE, .keep_all = TRUE) %>%
    dplyr::group_by(rarefyID) %>%
    dplyr::mutate(n_locations = dplyr::n_distinct(LONGITUDE,LATITUDE)) %>%
    dplyr::ungroup() %>%
    dplyr::filter(n_locations == 1) %>%
    dplyr::mutate(cell_extent = 0,
                  rarefyID_x = LONGITUDE,
                  rarefyID_y = LATITUDE) %>%
    dplyr::select(-LONGITUDE, -LATITUDE, -n_locations)

  ## join everything
  rarefyID_cell_centre <- dplyr::bind_rows(rarefyID_cell_centre,
                                            SL_coords, ML_coords) %>%
    dplyr::distinct(STUDY_ID, rarefyID, cell_extent, rarefyID_x, rarefyID_y)

  bt_grid <- bt %>%
    dplyr::select(CLIMATE, REALM, TAXA, StudyMethod, SAMPLE_DESC,
                  ABUNDANCE_TYPE, BIOMASS_TYPE, STUDY_ID, YEAR, PLOT,
                  cell, Species, DAY, MONTH, ABUNDANCE, BIOMASS, taxon,
                  resolution) %>%
    tidyr::unite(col = rarefyID, STUDY_ID, cell, sep = "_", remove = FALSE)
  k<-c(15,16)
  bt_grid[,k]<-apply(bt_grid[,k], 2, function(x) as.numeric(as.character(x)))

  return(bt_grid)
}
```

Resampling function to ensure no bias in sampling effort within studies, this is called by the runResampling() function

Parameter df = output from gridding function (bt_grid)

```
rarefysamples <- function(Year, SampleID, Species, currency, resamps) {
  # Checking arguments
  checkmate::assertSetEqual(length(Year), c(length(SampleID), length(Species), len
gth(currency)))

  minsample <- min(tapply(SampleID, Year, function(x) length(unique(x))))
```

```
  rareftab_list <- lapply( # beginning loop on repetitions
    X = seq_len(resamps),
    FUN = function(i) {
      selected_indices <- unlist(lapply( # beginning sub loop on years
        X = unique(Year),
        FUN = function(y) {
          samps <- unique(SampleID[Year == y])
          sam <- sample(samps, minsample, replace = TRUE)
          return(which(SampleID %in% sam & Year == y))
        })) # end of loop on years

      tYear      <- Year[selected_indices]
      tSpecies   <- Species[selected_indices]
      tcurrency <- currency[selected_indices]

      raref <- stats::aggregate(x = tcurrency, by = list(tYear, tSpecies), FUN = s
um)
      raref <- data.frame(i, raref)
      return(raref)

    }) # end of loop on repetitions

  rareftab <- do.call(rbind, rareftab_list)
  return(stats::setNames(rareftab, c("repeats", "Year", "Species", "currency")))

} # end of function


runResampling <- function(df) {

    TSrf <- list()
    rfIDs <- unique(df$rarefyID)

    for (i in 1:length(rfIDs)) {
      data <- df[df$rarefyID == rfIDs[i],]
      TSrf[[i]] <- rarefysamples(data$YEAR, data$SAMPLE_DESC, data$Species, data$A
BUNDANCE, 1)
    }
    names(TSrf) <- rfIDs

    rf <- do.call(rbind, TSrf)
    rf <- data.frame(rf, rfID = rep(names(TSrf), times = unlist(lapply(TSrf, nrow)
)))
    rf <- rf[!is.na(rf$Year),-1]
    rownames(rf) <- NULL

    rf1 <- rf %>%
      tidyr::separate(rfID, into =  c("STUDY_ID", "cell"), sep = "_", remove = F)
%>%
      dplyr::select(Year, Species, currency, rfID, STUDY_ID)
    colnames(rf1)<-c("Year", "Species", "Abundance", "rarefyID", "StudyID")

  return(rf1)
}

yrs<-rf1 %>% group_by(rarefyID) %>% summarise(yr=n_distinct(Year))
```

```
yrs20<-subset(yrs, yr>19)


rf1<-subset(rf1, rarefyID %in% yrs20$rarefyID)
```

Function to assign the temporal dynamics categories, this is called by the next block of code

```
getTrends<-function(x) {

  time <- rep("late",length(x))
  time[1:(round(length(x)/2))] <- "early"

  # tabulate f
  z_x <- table(x,time)

  tslen<-length(x)
  tssum<-sum(x)
  l<-c()

  for(k in 1:(tslen-1)){
    j<-abs(x[k+1]-x[k])
    l<-c(l, j)
    v<-sum(l)
  }

  bsline<-x[1]

  if(tslen>tssum) {

    rownames(z_x) <- c("absent","present")
    # get p value
    p_val <- chisq.test(z_x)$p.val

    # get early and late fractions
    f_early <- z_x["present","early"]/sum(z_x[,1])
    f_late <- z_x["present","late"]/sum(z_x[,2])

    if((f_early > f_late) & (p_val <= 0.05)) trend<--1
    if((f_early < f_late) & (p_val <= 0.05)) trend<-1
    if(p_val > 0.05) trend<-0
    runsPV<-runs.test(as.factor(x),alternative="less")
    runsTestPV<-runsPV$p.value
    if(runsTestPV<0.05) trendPlus<-10
    if(runsTestPV>0.05)trendPlus<-5
  }
  if(tslen==tssum) {
    p_val <- NA
    f_early <- NA
    f_late <- NA
    runsTestPV<-NA
    trend<-NA
```

```
    trendPlus<-NA
  }

  statSumm<-list("trendPlus"=trendPlus, "chiPval"=p_val, "runsPval"=runsTestPV,
                 "trend"=trend, "bsline"=bsline)
  return(statSumm)
}
```

Code to run the getTrends() function, this takes the output from the runResampling() function rf1 = TS

```
ids<-unique(TS$rarefyID)
df<-data.frame(rarefyID=0,Species=0,chiPval=0,
               trend=0,runsPval=0,trendPlus=0, bsline=0)
idplace<-1
for(id in ids){
  # getting data for relevant rarefyID
  data<-TS[TS$rarefyID==id,]
  data<- data[data$Abundance>0,]
  groups<-data.frame(as.character(data$Species),as.numeric(data$Year))
  data.mat<- tapply(data$Abundance,groups, FUN=sum)
  # formatting data into species by time matrix
  data.mat[is.na(data.mat)]<-0
  # removing species that are always absent
  numSp<-dim(data.mat)[1] # getting number of species
  df[idplace:(idplace+numSp-1),]<-cbind(rep(id,numSp),
                                        rownames(data.mat),rep(NA,numSp),rep(NA,nu
mSp),
                                        rep(NA,numSp),rep(NA,numSp),rep(NA,numSp),
                                        data.mat[,1],apply(data.mat,1,mean))


  bindf<- data.mat
  bindf[bindf>0]<-1

  bindf<-bindf[which(rowSums(bindf)<ncol(bindf)),]

  # create data frame to hold results
  if(!is.matrix(bindf)) bindf<-t(bindf)

  if(dim(bindf)[1]>0){
    # loop through the data
    for (i in 1:nrow(bindf)) {
      # Extract data for a species, do the new test, save output
      z<-bindf[i,]
      trn<-getTrends(z)
      df[df$rarefyID==id & df$Species==rownames(bindf)[i],
         3:7]<-c(trn$chiPval, trn$trend, trn$runsPval, trn$trendPlus,
                 trn$bsline)
    }
  }
  print(id)
  data.mat<-t(apply(data.mat,1,scale))
  idplace<-idplace+numSp
}
```

Combine the proportions of each temporal dynamics category with each assemblage, this is done where df is the output from the code block above

```
dft<-df[1:7]

dfspN<-as.data.frame(dft %>%
                       mutate(catClass=case_when(
                         trend==1 & bsline==0 ~ "Increasing",
                         trend==1 & bsline==1 ~ "Increasing",
                         trend==-1 & bsline==0 ~ "Decreasing",
                         trend==-1 & bsline==1 ~ "Decreasing",
                         is.na(trend) ~ "Always present",
                         trend==0 & trendPlus==10 ~ "Recurrent",
                         trend==0 & trendPlus==5 ~ "Random"
                       )
                       )
)

## combine with meta data

classMeta<-merge(dfspN, meta, by="rarefyID")

getRFsp<-as.data.frame(dfspN %>% group_by(rarefyID) %>%
                         summarise(sp=n_distinct(Species)))
getCl<-as.data.frame(dfspN %>% group_by(rarefyID, catClass) %>%
                       summarise(spCl=n_distinct(Species)))

doClProp<-merge(getCl, getRFsp, by="rarefyID")
doClProp$propCl<-doClProp$spCl/doClProp$sp

getProps<-as.data.frame(pivot_wider(doClProp, names_from=catClass,
                                    values_from=propCl))
getProps[is.na(getProps)]<-0
getPropsSp<-merge(getProps, getRFsp, by="rarefyID")

getPropMeta<-merge(getProp1, meta, by="rarefyID")
```

Model used in the first part of the analyses - output from code chunk above - getPropMeta, which is prepared using the DR_data function in the DirichletReg package

```
prop_data<-subset(getPropsSp, REALM!="Freshwater")



## prepare data
## select simplified response categories, apply recommended transformation for 0's
and 1's

dr_dat<-DR_data(prop_data[, c(6:8,20:21)])  %>%
  matrix(nrow=nrow(prop_data), byrow = FALSE)

colnames(dr_dat)<-c('Increasing', 'NoChange', 'Random', 'Recurrent',
                    'Decreasing')
```

```
dr_dat<-dr_dat %>% as_tibble() %>% mutate(realm = categ_data[,'REALM'],
         STUDY_ID = categ_data[,'STUDY_ID'],
         rarefyID = categ_data[,'rarefyID'])

bind <- function(...) cbind(...)

fitb<-brm(bind(Increasing, NoChange, Random, Recurrent, Decreasing) ~ 0 + realm +
(1 | STUDY_ID/rarefyID),
          data=dr_dat,
          family=dirichlet(refcat="Random"),
          cores=2, chains=4, iter = 4000)
```

Model used in the second part of the analyses, merge the output from above with the downloaded IUCN red list data

iucnsp = Red list data

```
colnames(dfspN)[colnames(dfspN) == 'valid_name'] <- 'scientificName'

spsIUCN<-merge(dfspN, iucnsp, by="scientificName", all=T)

spsIUCN_data<-select(spsIUCN, rarefyID, scientificName, redlistCategory,
                     Species, catClass, TAXA, CLIMATE, REALM)

bioIUCN<-spsIUCN_data %>%

  filter(redlistCategory != "Data Deficient") %>%
  filter(redlistCategory != "NA") %>%

  ## add numerical category
  mutate(redlist_numeric=ifelse(redlistCategory=="Least Concern", 1,
            ifelse(redlistCategory== "Lower Risk/least concern", 1,
              ifelse(redlistCategory=="Near Threatened", 2,
                ifelse(redlistCategory=="Lower Risk/near threatened", 2,
                  ifelse(redlistCategory=="Vulnerable", 3, 4)))))) %>%

  ## add simplified risk categories
  mutate(IUCN_risk=ifelse(redlist_numeric == 1, 1, 2)) %>%

  ## to get STUDY_ID
  separate(., rarefyID, into= c("STUDY_ID", "cell"), sep="_", remove=F)


## re-order levels
bioIUCN$catClass=factor(bioIUCN$catClass,
          levels=c("Random", "Always present", "Recurrent", "Decreasing", "Increas
ing"))

fitl<-lmer(redlist_numeric ~ turnoverCat * REALM + (1 | rarefyID),
              data=bioIUCN, REML=F)
```

The models shown here are those illustrated in the main text, to run those shown in the supplementary materials filter the data accordingly before running.
