# Supplementary material for: Linking species local trends from assemblage monitoring to global extinction risk
Source: Nat Commun. 2026 Jun 23;17:5071. doi: 10.1038/s41467-026-74132-7 (PMC13291242; doi:10.1038/s41467-026-74132-7)
Supplement: Supplementary file 5 — Transparent Peer Review file [file 41467_2026_74132_MOESM5_ESM.pdf]

nature portfolio

# Peer Review File

# Linking species local trends from assemblage monitoring to global extinction risk

Corresponding Author: Dr Faye Moyes

**This file contains all reviewer reports in order by version, followed by all author rebuttals in order by version.**

Version 0:

Reviewer comments:

Reviewer #1

(Remarks to the Author)
This paper examines the temporal dynamics of marine and terrestrial assemblages that have been sampled for at least 20 years and links these dynamics to the extinction risk of the focal species. The authors aim to determine whether the temporal trend of population change in a local assemblage could serve as an early-warning indicator of a species' global extinction risk. The results indicated that a higher extinction risk was associated with a greater frequency of decreasing local prevalence, and conversely, an increasing prevalence was linked to a lower extinction risk. Therefore, changes in a species' local prevalence can be used to predict its future threat status.

This study presents a straightforward approach to addressing a clearly defined question regarding the representativeness of assemblage-level monitoring data in assessing the threat status of global species. The work presents opportunities for species conservation applications, especially for those with long-term population datasets that face a high extinction risk on a global scale. However, some analyses are basic and potentially problematic or misleading, and there are several caveats that need to be resolved or clarified throughout to facilitate an accurate evaluation of species trends and threat status.

Major comments:
I appreciate the authors' use of long-term survey data to analyze population trends of species. However, an important issue arises from the fact that species survey data often cover different time periods, some of which may predate the IUCN's assessments of the species. For instance, when I searched for species data in BioTIME, I discovered that the cutoff dates for different data collections included the 1970s, 1980s, 2000s, and 2010s. While these historical data may provide some insight, the IUCN assessments typically rely on more recent data on population size and species range size (three generations or ten years). Hence, can the lagged population trends effectively reflect the present threat status of a species?

Measuring the threat status of a species requires an assessment of its global population size and trends. This study considers population data from various field studies; however, a significant challenge is that field data often focus on the population dynamics of a species within specific regions. First, seasonal environmental changes and migration can influence the population size of species in a region. This phenomenon is especially pronounced in birds and fish. Furthermore, some species have extensive distribution ranges, and their populations may increase in some areas while declining in others. This pattern is commonly observed in IUCN assessments (e.g., marine turtles, https://www.iucn-mtsg.org/statuses). The author should provide additional information to clarify whether the selected population data accurately represents the dynamic patterns of all populations of the species.

As shown in Figure 3, the author found that species with increasing population trends tend to have Least Concern (LC) status. However, the majority of species with declining population sizes also hold LC status, a trend that is more pronounced among terrestrial species. So, we can probably conclude that population decline and species becoming threatened is a low-probability event, especially considering that most species—whether declining, increasing, or fluctuating—exhibit a relatively low risk of extinction.

Finally, I would like to point out that the IUCN uses five criteria to assess the threat status of species, including population decline, small distribution range, small population size, etc. In recent years, a decreasing proportion of species has been classified as threatened based on population decline, highlighting the challenges associated with collecting accurate population data for species. Conversely, the majority of species are assessed as threatened due to their small distribution

range. Therefore, it suggests that relying solely on population decline data may not accurately reflect their true threat status. Therefore, the author needs to clarify in their study which species are classified as threatened based on distribution range and which are classified based on population trends. I personally believe that the IUCN Red List assessment methods require updating, while relying exclusively on population trends is insufficient.
Edgar, G. J. (2025). IUCN Red List criteria fail to recognise most threatened and extinct species. Biological Conservation, 301, 110880.

(Remarks on code availability)

Reviewer #2

(Remarks to the Author)
I enjoyed reading this manuscript, which investigates whether extinction risk based on the IUCN Red List correlates with population trends identified from a large database of monitoring efforts, covering both terrestrial and marine species. The work represents a substantial and important contribution to the field and the topic is not easy to quantify. The data are mostly from temperate latitudes, which should be made clear since the analytical question may be even more urgent for the speciose tropics, but this well-known data bias is unlikely to change in the future, maintaining the importance of the manuscript. The analysis is well conducted, from my perspective, but descriptions should be made more clear, straight-forward, and less evasive, particularly concerning the weaknesses in the data, where honesty and confidence would be better strategies. My suggestions are therefore minor and, pending their consideration by the authors, I recommend publication.

##General
The reader is not made aware early on or clearly about the weaknesses of the data (or of biases in the coverage of IUCN categories) i.e. is this truly a global cross-taxa relationship or are there weak points that need investigating further to confirm this? e.g. where are data deficient categories most prevalent? Perhaps other studies have covered this sufficiently that the reader can be referred to?
-e.g. "that is detectable across ecological realms and major taxonomic groups". Some major taxonomic groups...
-are marine realm only fish? also some corals? "marine invertebrates" is a very vague and diverse description. In the methods, "Thus, for this second analysis we mainly included data for vertebrates, although there were also limited matches for plants, crustaceans and molluscs." I would prefer this to be clear in the main text
-It should be stated that tropical assemblages are nearly absent (please confirm or contest) from the data. Fig. S2 makes it look like the "all" and "temperate only" results are nearly identical. I see this as a temperate rather than global study. Given that tropical species may be adapted to more stable conditions, perhaps temporal population change there may be more strongly linked to extinction risk? Furthermore, tropical populations may be more likely to be declining (e.g. Finn et al. 2023)? If there are (sub)tropical assemblages, it might be more interesting to analyse them alone, rather than temperate assemblages alone (i.e. focussing on the data weaknesses rather than their strengths)

Data bias issues are mentioned in the text e.g. "taxonomic biases are pervasive", but often using language that I find somewhat evasive, sometimes seemingly prioritising eloquence at the expense of clarity. I know this point is purely stylistic, but I encourage the authors to consider if there are more straight-forward ways to express things throughout the MS. If there are, clarity would be preferred in all cases. In particular, readers for whom English is not their mother-tongue may find the language clever but difficult to penetrate. Here, however, it is important to be candid about the shortcomings of the data so further studies know where to target

Extinction risk via IUCN is linked to population trends from data from the published literature, but isn't this partly how the IUCN define extinction risk for a species? e.g. "A temporal trend of decreasing prevalence in a local assemblage could be an early-warning indicator of a species global extinction risk 5,13." I would like the authors to assess any degree of circular reasoning here e.g. how likely are particular published data to be used for both the IUCN extinction risk and the temporal trends in this study? In case of worries, the argument could also be inverted as a validity check, i.e. if both the IUCN risk assessments and the temporal trends both used ALL available data (ideal scenario), we should see a perfect very high match. Then it would be good to know which species groups are weakest in the relationship, in order to improve process. Metrics that are independent from the IUCN could also be discussed to broaden the context e.g. fossil-derived risk baselines, discussed below. I wish to emphasise that I am happy to be wrong here, and at any rate I do not think the authors will have any problems dealing with this issue. But the issue should be confidently unpacked and advanced on.

##Abstract
How well do these populations monitored by the IUCN represent unmonitored of data deficient taxa within the same assemblage? This links to a comment below on the clarity of data representativeness. Here it could be clearer to mention that the populations monitored and thus the species tested were from temperate latitudes, potentially leaving tropical taxa unexplored in this sense

##Introduction
It would be good to mention how IUCN extinction risk does not always correlate well with other estimates of extinction risk e.g. Raja et al. 2021 for corals: https://doi.org/10.1111/geb.13321

The last sentence of the first paragraph is a bit dense and I suggest unpacking it into at least two sentences e.g. what precisely is meant by "representative and accurate"

"We anticipated that most species would exhibit random change in their temporal occurrence, with only a small proportion of populations showing either decreasing or increasing temporal prevalence 27". One would also expect some species to randomly demonstrate decreasing or increasing trends as part of a random distribution. How significant is the number identified? I would be more comfortable with an earlier clarification of which probability level is used/deemed acceptable to decide random from non-random change

L118. "These results suggest that temporal prevalence patterns previously reported for... ". A simple description of the previously reported patterns would be useful here e.g. "These results suggest that decreasing temporal prevalence patterns previously reported for... "

"global extinction risk". "IUCN-based global extinction risk" would seem more accurate

"Specifically, species with higher extinction risk were more likely to have populations with decreasing prevalence". Some effect sizes and confidence intervals would be nice here. In general, the manuscript focusses so far on significance but I would be interested to read about the effect size and how the authors interpret it e.g. is it low because of data deficiency, because of the nature of the IUCN categories, or because of the temporal scale (extent covered or resolution)?

"Previous work 27 found that species with decreasing and increasing temporal prevalence are influential in driving compositional change at the assemblage level, ". This sounds like circular reasoning. Maybe a line explaining how circularity was avoided in that case

"Long term decreases in species prevalence may be harbingers of extinction events 13,39.". Previous extinction events were quantified over 1000s to millions of years, so appropriate studies are needed for context. The influence of geographical range size (Payne & Finnegan 2007) and occupancy trajectories (Kiessling & Kocsis 2015) on fossil extinction risk appear relevant. Links between temporal prevalence here extending to extirpation and global extinction have also been made to a population's position within its species thermal niche during climate change e.g. Reddin et al. 2025 as an application of thermal bias in Stuart-Smith et al. 2015. That approach may link well to the paragraph beginning, "Our results have direct implications for conservation and management", especially linking to climate change.

Payne, J. L., & Finnegan, S. (2007). The effect of geographic range on extinction risk during background and mass extinction. Proceedings of the National Academy of Sciences, 104(25), 10506-10511.
Kiessling, W., & Kocsis, Á. T. (2016). Adding fossil occupancy trajectories to the assessment of modern extinction risk. Biology letters, 12(10), 20150813.
Reddin, C. J., Landwehrs, J. P., Mathes, G. H., Ullmann, C. V., Feulner, G., & Aberhan, M. (2025). Marine species and assemblage change foreshadowed by their thermal bias over Early Jurassic warming. Nature Communications, 16(1), 1370.
Stuart-Smith, R. D., Edgar, G. J., Barrett, N. S., Kininmonth, S. J., & Bates, A. E. (2015). Thermal biases and vulnerability to warming in the world’s marine fauna. Nature, 528(7580), 88-92.

Why are seemingly so few species identified here as “declining” versus so many elsewhere e.g. in Finn et al. 2023? Is it due to differences in the geographical foci of the data?

L258. "By alleviating data caveats". Unclear what this means

"the full spectrum of temporal prevalence dynamics". Surely five categories cannot be considered a full spectrum, following the analogy of light? Full range, perhaps. Or is this what the authors meant?

"may even be regulated in the statistical sense...". A comma here may improve clarity: "may even be regulated, in the statistical sense..."

L271. "linkages between two approaches that ". Which are these two approaches, explicitly?

Fig. 1. For clarity, consider putting "%" on the x-axis for C and D.

Fig. 2. "). The estimated values from a mixed effect model are shown in Fig. S2 (Table S1)." Values of what?

##Methods
307. I am not clear on the methodological separation between recurrent and random change categories

“…included a random effect nesting assemblages within studies to account for the structure of the data”. I wonder how the authors feel about adding species as a random effect, given that each species may have many populations, comprising the data. If this creates problems, it might be good to know whether some species are particularly influential to the observed relationships, and, if so, whether such species have shared characteristics or are a random subset.

At the end of the methods, please add a definition for "temperate" to remove ambiguity

Fig. S2. When presenting proportions, I would like to know the total numbers involved somewhere in the figure, for instance in the caption

Fig. S7. It would be nice to have the p-values as exact values rather than which threshold they pass

Please add line numbers to manuscripts for peer review in the future. Otherwise the review was a pleasure.

(Remarks on code availability)

Version 1:

Reviewer comments:

Reviewer #1

(Remarks to the Author)
This study is valuable for its approach in linking local population dynamics of species to global extinction risk using assemblage-level monitoring data. While the authors have addressed some of the issues I previously raised, many fundamental problems remain unresolved. My additional comments are as follows:

The authors main conclusion that higher extinction risk was associated with a higher frequency of decreasing local prevalence, and vice-versa for increasing prevalence, in fact, already incorporated into Criteria A and C of the IUCN assessment framework. As a matter of fact, the measurement of population trends in the IUCN framework is to some extent more complex than the method adopted by the authors. The authors work merely restates a result that is already well-known to us.

Secondly, the authors main results are inconsistent with their own claims. As can be seen from Figure 3, for taxa in different regions, nearly all species with increasing or decreasing populations are classified as "Least Concern." This intuitively demonstrates that a species population trend cannot reflect its threatened status. Furthermore, nearly all species with randomly fluctuating or stable populations are also "Least Concern"—this finding is disconnected from the authors' core argument that "population trends can improve extinction risk prediction."

On the other hand, the authors research results may be subject to survivorship bias. It can also be observed that the majority of taxa studied by the authors are "Least Concern" species. This proportion may be as high as 95% among terrestrial species. Such sampling bias could lead to the authors results primarily relying on differences between "Least Concern" and "Near Threatened/Vulnerable" species, while lacking valid verification for high-risk species.

The authors main analysis focuses on 973 species; however, there is a significant imbalance in geographic and taxonomic coverage. For instance, the study concentrates on Europe and North America, while other regions—tropical areas in particular—are not included. This bias means the conclusions may only apply to temperate vertebrates and cannot be extended to tropical ecosystems or more vulnerable taxa like invertebrates, significantly limiting the study's global conservation relevance.

Additionally, I still hold the view that population research at the regional scale cannot represent the overall situation. In their response, the authors stated that the IUCN also conducts regional-scale endangered risk assessments. However, for many species with wide regional distributions, regional-scale assessment results may be invalid or even contradictory to the overall situation. For example, some species are generally safe at the global level but show a declining trend in a specific region. We cannot directly combine these two sets of results (regional trends and global status).

In the Methods section, the authors stated: "To ensure that our results were not overly influenced by the predominance of 'Least Concern' species, we further simplified the extinction risk categories into only two groups: 'low risk' (corresponding to 1 in the above classification) and 'high risk', combining the remaining categories (2 to 4)." However, I believe that classifying "Near Threatened" species as "high-risk" constitutes a misunderstanding of IUCN guidelines.

Should the authors consider using changes in population abundance, rather than the presence or absence of a species within a community, as the criterion for determining population trends?

(Remarks on code availability)

Reviewer #2

(Remarks to the Author)
The authors have satisfied my previous, minor suggestions and I highly recommend the manuscript for publication in nature Communications. I thank the authors for a pleasant review and wish them all the best.

(Remarks on code availability)

Version 2:

Reviewer comments:

Reviewer #1

(Remarks to the Author)
I am satisfied with the author's response. This paper provides significant support for accurately predicting extinction risk based on species' local trends. The quality of the paper reflects the journal's high standards for selecting top-tier research.

(Remarks on code availability)

**Open Access** This Peer Review File is licensed under a Creative Commons Attribution 4.0 International License, which permits use, sharing, adaptation, distribution and reproduction in any medium or format, as long as you give appropriate credit to the original author(s) and the source, provide a link to the Creative Commons license, and indicate if changes were made.
In cases where reviewers are anonymous, credit should be given to 'Anonymous Referee' and the source.
The images or other third party material in this Peer Review File are included in the article's Creative Commons license, unless indicated otherwise in a credit line to the material. If material is not included in the article's Creative Commons license and your intended use is not permitted by statutory regulation or exceeds the permitted use, you will need to obtain permission directly from the copyright holder.
To view a copy of this license, visit https://creativecommons.org/licenses/by/4.0/

**Response letter for NCOMMS-24-75253**

Thank you again for submitting your manuscript "Assemblage-level monitoring provides insights into extinction risk" to Nature Communications. We have now received reports from 2 reviewers and, after careful consideration, we have decided to invite a major revision of the manuscript.

As you will see from the reports copied below, the reviewers raise important concerns. We find that these concerns limit the strength of the study, and therefore we ask you to address them with additional work. Without substantial revisions, we will be unlikely to send the paper back to review. In particular, concerns raised about data analysis and potentially bias must be addressed.

If you feel that you are able to comprehensively address the reviewers' concerns, please provide a point-by-point response to these comments along with your revision. Please show all changes in the manuscript text file with track changes or colour highlighting. If you are unable to address specific reviewer requests or find any points invalid, please explain why in the point-by-point response.

We hope to receive your revised paper within three months, but we understand that revisions may take longer. Please let us know if you find that the revision process will take substantially more time.

Thank you for the opportunity to revise our paper.

Please note that we have submitted two documents for the revised main text: one with the in-line tracked changes highlighted and one version without any highlighted edits. The line numbers mentioned in our response are now corrected for the clean version.

REVIEWER COMMENTS

Reviewer #1 (Remarks to the Author):

This paper examines the temporal dynamics of marine and terrestrial assemblages that have been sampled for at least 20 years and links these dynamics to the extinction risk of the focal species. The authors aim to determine whether the temporal trend of population change in a local assemblage could serve as an early-warning indicator of a species' global extinction risk. The results indicated that a higher extinction risk was associated with a greater frequency of decreasing local prevalence, and conversely, an increasing prevalence was linked to a lower extinction risk. Therefore, changes in a species' local prevalence can be used to predict its future threat status.

This study presents a straightforward approach to addressing a clearly defined question regarding the representativeness of assemblage-level monitoring data in assessing the threat status of global species. The work presents opportunities for species conservation applications, especially for those with long-term population datasets that face a high extinction risk on a global scale. However, some analyses are basic and potentially problematic or misleading, and there are several caveats that need to be resolved or clarified throughout to facilitate an accurate evaluation of species trends and threat status.

Response: Thank you for your thoughtful review of our manuscript. We agree this is a straightforward approach to improve assessments of threat status that uses simple and robust methods, aimed to deal also with the structured nature of the data. We have improved the text to clarify the data caveats (as also recommended by Reviewer #2) and to better describe the approach we implemented. We trust

these changes have strengthened the manuscript and removed any potential misleading statements, and would welcome any concrete advice to improve the analyses if the Reviewer would still think any modifications would be necessary to reassure them the results are robust.

Major comments:

I appreciate the authors' use of long-term survey data to analyze population trends of species. However, an important issue arises from the fact that species survey data often cover different time periods, some of which may predate the IUCN's assessments of the species. For instance, when I searched for species data in BioTIME, I discovered that the cutoff dates for different data collections included the 1970s, 1980s, 2000s, and 2010s. While these historical data may provide some insight, the IUCN assessments typically rely on more recent data on population size and species range size (three generations or ten years). Hence, can the lagged population trends effectively reflect the present threat status of a species?

Response: Thank you for this thoughtful comment. It is indeed the case that survey data may cover different periods compared to IUCN assessments. However, there are a number of recognised caveats and concerns around how IUCN criteria are defined and implemented, including the data used for estimating extinction risk and whether current classifications are able to capture the *true* status of species [1-4]. One such critical issue is the arbitrary choices of a 10-year window (or three generations) and baseline year, which can cause classifications to vary across extinction risk categories, and lead to erroneous classifications, e.g. if a decline or increase has occurred before the focal 10-y period or due to rapid population changes going unrecognized [4,5]. On the other hand, using longer time series when they are available is recommended, as they provide more accurate prediction of trends and extinction risk [1-3]. The lack of periodic re-assessments for the majority of species is also recognized as a key caveat for the IUCN classification, leading to high risk of species being misclassified [2]. As such, having data preceding any baseline year chosen for IUCN assessments or covering longer periods is key to more reliably estimating population trends, and thus we see this a major strength in our analysis.

We emphasise that our intention is not to replicate or replace the IUCN assessments and criteria, but rather to complement and improve the extinction assessments, and we believe that our approach has several key strengths. First, using longer time series which give more robust insights into any type of change taking place. Second, using assemblage monitoring data allows estimating temporal trends for many more species, thus contributing to alleviate taxonomic and system biases in IUCN assessments [2,4,6]. Third, by illustrating how other types of monitoring data and analysis can provide alternative sources of data and estimates when population-level monitoring may not be available to estimate current criteria, e.g. leading to a high number of species being classified as Data Deficient [2,7].

We have now added an extensive section to the Discussion to more clearly articulate these points in the revised manuscript (lines 256-272).

Measuring the threat status of a species requires an assessment of its global population size and trends. This study considers population data from various field studies; however, a significant challenge is that field data often focus on the population dynamics of a species within specific regions. First, seasonal environmental changes and migration can influence the population size of species in a region. This phenomenon is especially pronounced in birds and fish. Furthermore, some

species have extensive distribution ranges, and their populations may increase in some areas while declining in others. This pattern is commonly observed in IUCN assessments (e.g., marine turtles, https://www.iucn-mtsg.org/statuses). The author should provide additional information to clarify whether the selected population data accurately represents the dynamic patterns of all populations of the species.

Response: The IUCN framework includes global, regional and national (re)assessments, and as pointed out there can be variation in population trends between regions. Identifying whether the populations as defined in our study "accurately represents the dynamic patterns of all populations of the species" would be a major task well beyond the scope of the current analysis, and which would likely be unfeasible for many species in our data for which there is poor or no information on their ranges. But we agree this is a key aspect to consider, as we had already emphasised that different populations would fall into different categories of temporal dynamics. In the revised manuscript, we now more clearly discuss spatial variation in population trends (lines 224-227), we included an example mentioning this pattern among regions (lines 349-351), and we also now explicitly indicate that we did not assess the representativeness of the populations regarding temporal dynamics across species global ranges (lines 346-349 in the Methods).

As shown in Figure 3, the author found that species with increasing population trends tend to have Least Concern (LC) status. However, the majority of species with declining population sizes also hold LC status, a trend that is more pronounced among terrestrial species. So, we can probably conclude that population decline and species becoming threatened is a low-probability event, especially considering that most species—whether declining, increasing, or fluctuating—exhibit a relatively low risk of extinction.

Response: We agree with the reviewer and have added to our sentence in lines 169-170: "suggesting that extinction risk in general may be of low probability for the species in our data".

Finally, I would like to point out that the IUCN uses five criteria to assess the threat status of species, including population decline, small distribution range, small population size, etc. In recent years, a decreasing proportion of species has been classified as threatened based on population decline, highlighting the challenges associated with collecting accurate population data for species. Conversely, the majority of species are assessed as threatened due to their small distribution range. Therefore, it suggests that relying solely on population decline data may not accurately reflect their true threat status. Therefore, the author needs to clarify in their study which species are classified as threatened based on distribution range and which are classified based on population trends. I personally believe that the IUCN Red List assessment methods require updating, while relying exclusively on population trends is insufficient.

Edgar, G. J. (2025). IUCN Red List criteria fail to recognise most threatened and extinct species. Biological Conservation, 301, 110880.

Response: We agree with the Reviewer's points, and believe "the challenges associated with collecting accurate population data for species" is a great argument for supporting efforts to leverage assemblage monitoring data towards improving extinction risk assessments and classifications. We also note that while we focused on temporal prevalence dynamics, assemblage time series data can also contribute to assess changes in species distributions, e.g. by tracking colonization and extirpation

events in a more spatially explicit way. Estimates of species range sizes, whilst perhaps simpler to produce, do not always give an accurate picture of how well a population is doing, or might miss varying trends across the species range. To address this comment, we have clearly indicated in the Introduction that IUCN classification relies on several criteria (lines 62-63) and that the application of criteria can lead to variability in threat status estimates (lines 69-71), having added relevant references e.g., [3,4]. In addition, we added several key points on how our approach can help improve threat status assessments in the Discussion (lines 256-271), while noting that the focus of our paper is not on directly evaluating the current IUCN criteria.

Reviewer #2 (Remarks to the Author):

I enjoyed reading this manuscript, which investigates whether extinction risk based on the IUCN Red List correlates with population trends identified from a large database of monitoring efforts, covering both terrestrial and marine species. The work represents a substantial and important contribution to the field and the topic is not easy to quantify. The data are mostly from temperate latitudes, which should be made clear since the analytical question may be even more urgent for the speciose tropics, but this well-known data bias is unlikely to change in the future, maintaining the importance of the manuscript. The analysis is well conducted, from my perspective, but descriptions should be made more clear, straight-forward, and less evasive, particularly concerning the weaknesses in the data, where honesty and confidence would be better strategies. My suggestions are therefore minor and, pending their consideration by the authors, I recommend publication.

Response: Thank you for your thoughtful review of our manuscript, and we are happy to hear you enjoyed reading it. We have addressed all the issues raised, including being more upfront about the data caveats and simplifying language.

##General

The reader is not made aware early on or clearly about the weaknesses of the data (or of biases in the coverage of IUCN categories) i.e. is this truly a global cross-taxa relationship or are there weak points that need investigating further to confirm this? e.g. where are data deficient categories most prevalent? Perhaps other studies have covered this sufficiently that the reader can be referred to?

-e.g. "that is detectable across ecological realms and major taxonomic groups". Some major taxonomic groups...

-are marine realm only fish? also some corals? "marine invertebrates" is a very vague and diverse description. In the methods, "Thus, for this second analysis we mainly included data for vertebrates, although there were also limited matches for plants, crustaceans and molluscs." I would prefer this to be clear in the main text

-It should be stated that tropical assemblages are nearly absent (please confirm or contest) from the data. Fig. S2 makes it look like the "all" and "temperate only" results are nearly identical. I see this as a temperate rather than global study. Given that tropical species may be adapted to more stable conditions, perhaps temporal population change there may be more strongly linked to extinction risk? Furthermore, tropical populations may be more likely to be declining (e.g. Finn et al. 2023)? If there

are (sub)tropical assemblages, it might be more interesting to analyse them alone, rather than temperate assemblages alone (i.e. focussing on the data weaknesses rather than their strengths)

Data bias issues are mentioned in the text e.g. "taxonomic biases are pervasive", but often using language that I find somewhat evasive, sometimes seemingly prioritising eloquence at the expense of clarity. I know this point is purely stylistic, but I encourage the authors to consider if there are more straight-forward ways to express things throughout the MS. If there are, clarity would be preferred in all cases. In particular, readers for whom English is not their mother-tongue may find the language clever but difficult to penetrate. Here, however, it is important to be candid about the shortcomings of the data so further studies know where to target

Response: We have implemented changes throughout the manuscript to address these points. Specifically, we now clearly indicate that most of our data are from temperate locations (including in the Abstract, line 41), and we added detailed caveats rather than only mentioning “taxonomic and geographic biases”, e.g. explicitly mentioning the absence of amphibian and tropical species, highlighting these are two groups with high extinction risk (lines 281-282). We also modified the sentences referring to taxonomic groups as suggested (lines 91-93 and 208), and we revised the manuscript to only use the word “global” when referring to the IUCN “global” classification of extinction risk. We trust these changes now clearly indicate the caveats and biases in the data and have also improved communication overall.

Extinction risk via IUCN is linked to population trends from data from the published literature, but isn't this partly how the IUCN define extinction risk for a species? e.g. "A temporal trend of decreasing prevalence in a local assemblage could be an early-warning indicator of a species global extinction risk 5,13." I would like the authors to assess any degree of circular reasoning here e.g. how likely are particular published data to be used for both the IUCN extinction risk and the temporal trends in this study? In case of worries, the argument could also be inverted as a validity check, i.e. if both the IUCN risk assessments and the temporal trends both used ALL available data (ideal scenario), we should see a perfect very high match. Then it would be good to know which species groups are weakest in the relationship, in order to improve process. Metrics that are independent from the IUCN could also be discussed to broaden the context e.g. fossil-derived risk baselines, discussed below. I wish to emphasise that I am happy to be wrong here, and at any rate I do not think the authors will have any problems dealing with this issue. But the issue should be confidently unpacked and advanced on.

Response: To the best of our knowledge, we are unaware of IUCN risk assessments using monitoring data at the assemblage/community-level, and therefore it is highly unlikely that any data has been used for both our estimates of temporal dynamics and for IUCN assessments. On the other hand, IUCN assessments rely on five quantitative criteria related to population size, trend, and distribution that are implemented and validated by dedicated expert-led groups. Indeed, we believe our approach can provide alternative sources of data to improve assessments of extinction risk, including as suggested via validation with independent data. We now include a clear indication that IUCN classification relies on quantitative criteria (lines 62-63) and we more clearly outline the key strengths of our approach using long-term data assemblage-level data to estimate temporal prevalence in the Discussion (lines 267-271) - noting e.g. that it can contribute to alleviating some critical issues with how IUCN criteria are implemented using arbitrary and rigid rules, such as population trends being

“measured over the longer of 10 years or 3 generations” (see also our response to Reviewer #1 above regarding IUCN criteria).

##Abstract

How well do these populations monitored by the IUCN represent unmonitored of data deficient taxa within the same assemblage? This links to a comment below on the clarity of data representativeness. Here it could be clearer to mention that the populations monitored and thus the species tested were from temperate latitudes, potentially leaving tropical taxa unexplored in this sense

Response: Done - we added “mostly from temperate regions” (line 41).

##Introduction

It would be good to mention how IUCN extinction risk does not always correlate well with other estimates of extinction risk e.g. Raja et al. 2021 for corals: https://doi.org/10.1111/geb.13321

The last sentence of the first paragraph is a bit dense and I suggest unpacking it into at least two sentences e.g. what precisely is meant by "representative and accurate"

Response: We have restructured the last two sentences of the Introduction, and added the suggested reference to mention mismatches with other estimates of extinction risk (lines 69-71).

"We anticipated that most species would exhibit random change in their temporal occurrence, with only a small proportion of populations showing either decreasing or increasing temporal prevalence 27". One would also expect some species to randomly demonstrate decreasing or increasing trends as part of a random distribution. How significant is the number identified? I would be more comfortable with an earlier clarification of which probability level is used/deemed acceptable to decide random from non-random change

Response: We have clarified how the populations are assigned to the different categories of temporal prevalence both in the main text (lines 96-105) and in the Methods (lines 358-371). Specifically, the series of steps followed determine whether there is a statistically significant change in prevalence between an early and later period of the time series: if yes, then there is directional change, either increasing or decreasing. If not, then a population can show a pattern that is not statistically different from a random reshuffling of the sequence of presences and absences, thus being classified as random change *versus* recurrent change, where the sequence has unusually long periods of presence and absence, conditioned on the total number of presences or absences in the sequence. We used the common significance level of $p<0.05$ for each test (now added to line 363). We have added more details to the revised text and all the steps are explained in Gotelli et al. 2021, but we will be happy to add more information if needed.

For the first analysis in the manuscript, we then modelled the full vector of the proportion of populations in each category of temporal prevalence per assemblage, thus providing an assessment of the distribution of temporal prevalence dynamics.

L118. "These results suggest that temporal prevalence patterns previously reported for... ". A simple description of the previously reported patterns would be useful here e.g. "These results suggest that decreasing temporal prevalence patterns previously reported for... "

Response: We modified the sentence to clarify which patterns we were referring to, as suggested (lines 124-127).

"global extinction risk". "IUCN-based global extinction risk" would seem more accurate

Response: We agree and have now altered our text accordingly.

"Specifically, species with higher extinction risk were more likely to have populations with decreasing prevalence". Some effect sizes and confidence intervals would be nice here. In general, the manuscript focusses so far on significance but I would be interested to read about the effect size and how the authors interpret it e.g. is it low because of data deficiency, because of the nature of the IUCN categories, or because of the temporal scale (extent covered or resolution)?

Response: Thank you for this relevant suggestion. We have now added effect sizes for the models estimating the relationship between extinction risk and the observed temporal dynamics (Tables S3 to S6), and added the description to the Methods in lines 441-442. We note that confidence intervals were already reported in the initial version of the manuscript.

"Previous work 27 found that species with decreasing and increasing temporal prevalence are influential in driving compositional change at the assemblage level, ". This sounds like circular reasoning. Maybe a line explaining how circularity was avoided in that case

Response: Thank you for pointing this out. This sentence has been improved and moved to the Discussion (lines 302-306); this refers to a previous related study by Gotelli et al. 2021, where we showed that the species with increasing and decreasing temporal prevalence were the main drivers of changes in assemblage composition - that is, despite the low proportion of species showing directional change within assemblages, these are the ones underpinning long-term changes in composition. We hope this clarifies this point, as we believe there is no circularity.

"Long term decreases in species prevalence may be harbingers of extinction events 13,39.". Previous extinction events were quantified over 1000s to millions of years, so appropriate studies are needed for context. The influence of geographical range size (Payne & Finnegan 2007) and occupancy trajectories (Kiessling & Kocsis 2015) on fossil extinction risk appear relevant. Links between temporal prevalence here extending to extirpation and global extinction have also been made to a population's position within its species thermal niche during climate change e.g. Reddin et al. 2025 as an application of thermal bias in Stuart-Smith et al. 2015. That approach may link well to the paragraph beginning, "Our results have direct implications for conservation and management", especially linking to climate change.

Payne, J. L., & Finnegan, S. (2007). The effect of geographic range on extinction risk during background and mass extinction. Proceedings of the National Academy of Sciences, 104(25), 10506-10511.

Kiessling, W., & Kocsis, Á. T. (2016). Adding fossil occupancy trajectories to the assessment of modern extinction risk. Biology letters, 12(10), 20150813.

Reddin, C. J., Landwehrs, J. P., Mathes, G. H., Ullmann, C. V., Feulner, G., & Aberhan, M. (2025). Marine species and assemblage change foreshadowed by their thermal bias over Early Jurassic warming. Nature Communications, 16(1), 1370.

Stuart-Smith, R. D., Edgar, G. J., Barrett, N. S., Kininmonth, S. J., & Bates, A. E. (2015). Thermal biases and vulnerability to warming in the world's marine fauna. Nature, 528(7580), 88-92.

Response: Thank you for this insightful suggestion and for driving us to amend our use of "extinction events" in this instance. We have modified this sentence to "...future threat status and extirpation, and potentially global extinction" (lines 230-231), and we added a sentence regarding how the uncertainty around the true number of threatened species hampers estimates of current extinction rates (lines 234-236). In addition, we added a sentence in the Discussion highlighting the need for complementary estimates of extinction risk to address the current biodiversity crisis (lines 304-309).

Why are seemingly so few species identified here as "declining" versus so many elsewhere e.g. in Finn et al. 2023? Is it due to differences in the geographical foci of the data?

Response: We believe this may indeed be attributed to a few aspects related to the different types of data used. Finn et al. 2023 used population data from the IUCN database, which are highly skewed towards vertebrates and large species, whereas our assemblage-level data has a broader scope and did not include any amphibian species for instance, which tend to show high extinction risk and are characterised by declining populations (as shown by [8] and e.g., [9]). Overall, this last study using the Living Planet Database found that 15% of populations declined, 18% increased, and 67% showed no net changes over time. Also, our data also does not include tropical regions, where drivers of extinction risk may be more strongly affecting a high number of species, which in turn tend to have small ranges, another criteria leading to species being evaluated with a high threat status. As we emphasize in the manuscript, assessing the full range of temporal trends can provide important insights to better quantify patterns of change and improve the use of research and conservation resources.

L258. "By alleviating data caveats". Unclear what this means

Response: We have now added a new section indicating the key strengths of our approach to the Discussion (lines 256-272), one of each is that using assemblage monitoring data allows estimating temporal trends for many more species that may currently be unassessed or classified as Data Deficient, thus we believe the meaning of this sentence is now made clear (now in lines 267-261).

"the full spectrum of temporal prevalence dynamics". Surely five categories cannot be considered a full spectrum, following the analogy of light? Full range, perhaps. Or is this what the authors meant?

Response: We agree, thank you, indeed range was what we meant and we have now updated our text accordingly, e.g. line 294.

"may even be regulated in the statistical sense...". A comma here may improve clarity: "may even be regulated, in the statistical sense..."

Response: This sentence has been re-written and this bit of text has been deleted (now lines 301-302).

L271. "linkages between two approaches that ". Which are these two approaches, explicitly?

Response: We modified the sentence to clarify the approaches as "species-level extinction assessments and assemblage-level biodiversity analyses" (lines 310-311).

Fig. 1. For clarity, consider putting "%" on the x-axis for C and D.

Response: Done, we have updated this figure accordingly.

Fig. 2. "). The estimated values from a mixed effect model are shown in Fig. S2 (Table S1)." Values of what?

Response: This refers to the proportion values estimated from the model, we have updated the text to reflect this (line 151).

##Methods

307. I am not clear on the methodological separation between recurrent and random change categories

Response: We have now added text to clarify the distinction between the categories and more details about the tests implemented to classify species either as random or recurrent (lines 100-104 and 355-371; see also our response to your comment above).

"…included a random effect nesting assemblages within studies to account for the structure of the data". I wonder how the authors feel about adding species as a random effect, given that each species may have many populations, comprising the data. If this creates problems, it might be good to know whether some species are particularly influential to the observed relationships, and, if so, whether such species have shared characteristics or are a random subset.

Response: This comment refers to the first analysis in the manuscript - which we now more clearly identify under the heading "Patterns of temporal prevalence" in the main text. For this analysis, we modelled the vector of proportions of the temporal categories for each assemblage as a function of realm - i.e. in this case the records are not at the species/population level, but rather at the assemblage level indicating the percentage of populations for each of the five categories of temporal change. For the second analysis (now under the header "Relationship between extinction risk and local temporal dynamics") indeed we need to account for having different populations from the same species, and so

we included a random effect for assemblage to account for multiple species being sampled within each time series.

At the end of the methods, please add a definition for "temperate" to remove ambiguity

Response: We have now added a sentence explaining this (lines 449-453).

Fig. S2. When presenting proportions, I would like to know the total numbers involved somewhere in the figure, for instance in the caption

Response: We have now added the numerical proportions to the legend with total numbers of species' populations falling into each category to the legend.

Fig. S7. It would be nice to have the p-values as exact values rather than which threshold they pass

Response: Done.

Please add line numbers to manuscripts for peer review in the future. Otherwise the review was a pleasure.

Response: Sorry - we have now added line numbers, and thank you!

References


1 d'Eon-Eggertson, F., Dulvy, N. K. & Peterman, R. M. Reliable identification of declining populations in an uncertain world. *Conservation Letters* **8**, 86-96 (2015).

2 Cazalis, V. *et al.* Bridging the research-implementation gap in IUCN Red List assessments. *Trends in Ecology & Evolution* **37**, 359-370, doi:https://doi.org/10.1016/j.tree.2021.12.002 (2022).

3 Akçakaya, H. R. *et al.* Calculating population reductions of invertebrate species for IUCN Red List assessments. *Journal of insect conservation* **25**, 377-382 (2021).

4 Edgar, G. J. IUCN Red List criteria fail to recognise most threatened and extinct species. *Biological Conservation* **301**, 110880 (2025).

5 Fox, R. *et al.* Insect population trends and the IUCN Red List process. *Journal of Insect Conservation* **23**, 269-278 (2019).

6 Bachman, S. P. *et al.* Progress, challenges and opportunities for Red Listing. *Biological Conservation* **234**, 45-55, doi:https://doi.org/10.1016/j.biocon.2019.03.002 (2019).

7 Borgelt, J., Dorber, M., Høiberg, M. A. & Verones, F. More than half of data deficient species predicted to be threatened by extinction. *Communications Biology* **5**, 679, doi:10.1038/s42003-022-03638-9 (2022).

8 Finn, C., Grattarola, F. & Pincheira-Donoso, D. More losers than winners: investigating Anthropocene defaunation through the diversity of population trends. *Biological Reviews* **98**, 1732-1748, doi:https://doi.org/10.1111/brv.12974 (2023).

9 Daskalova, G. N., Myers-Smith, I. H. & Godlee, J. L. Rare and common vertebrates span a wide spectrum of population trends. *Nature Communications* **11**, 4394, doi:10.1038/s41467-020-17779-0 (2020).

**REVIEWER COMMENTS**

**Reviewer #1 (Remarks to the Author):**

This study is valuable for its approach in linking local population dynamics of species to global extinction risk using assemblage-level monitoring data. While the authors have addressed some of the issues I previously raised, many fundamental problems remain unresolved. My additional comments are as follows:

The authors main conclusion that higher extinction risk was associated with a higher frequency of decreasing local prevalence, and vice-versa for increasing prevalence, in fact, already incorporated into Criteria A and C of the IUCN assessment framework. As a matter of fact, the measurement of population trends in the IUCN framework is to some extent more complex than the method adopted by the authors. The authors work merely restates a result that is already well-known to us.

We apologise if we have not been able to clearly communicate the aims of our study, as it seems the Reviewer might be missing the overall point of our analysis. We do not aim to validate or test the IUCN classification or assessment criteria. Rather, we aim to ask whether temporal prevalence trends from long-term assemblage monitoring data with standardised sampling would reflect a signal consistent with IUCN extinction risk categories across species and habitats. We have aimed to further clarify the aims of the study (lines 43-47 in the abstract and lines 83-87, 99-103 in the text), as well as the implications of our results, particularly focusing on how they provide novel, quantitative complementary insights to improve biodiversity assessments and alleviate knowledge gaps (lines 299-306). We also modified the title to remove any undue expectation of *improving the IUCN classification or assessment criteria*.

Regarding the point that our results "that higher extinction risk was associated with a higher frequency of decreasing local prevalence, and vice-versa for increasing prevalence" … "merely restates a result that is already well-known to us". Given that we use assemblage-level data in our study, it was by no means obvious that this would be the case, and we believe most ecologists would not have predicted such a concordance. Thus, while we agree that identifying a link between decreasing temporal prevalence and higher extinction risk (and vice versa for increasing temporal prevalence) matches *logical expectations*, we disagree that our results are "already well-known". For instance, several independent studies using long-term assemblage monitoring data have shown an overall balance in richness and abundance trends (e.g., [1-4]). Moreover, a balance among species trends can also be observed, as evidenced even when analysing LPI data, despite important differences between taxa (e.g., [5-10]). We had already indicated this mix of trends in the previous version, and have now revised the text to maximise clarity on these points.

On the other hand, a mismatch between species trends and IUCN extinction risk categories and the lack of appropriate temporal data for assessing trends are known issues (e.g. [11-14]). Thus, while we agree that the measurement of population trends in the IUCN framework is

more “complex” than evaluating temporal occupancy trends, our novel analysis provides relevant and urgently needed insights to improve any type of analysis needed to support conservation efforts.

Overall, our results support the validity of using adequate assemblage-level monitoring as a useful tool for e.g. identifying species currently not included in the IUCN assessments as potentially at risk, while further revealing the complex links between temporal and extinction risk dynamics. Such analyses can provide invaluable and complementary quantification of species spatio-temporal patterns that can be useful for improving and/or validating extinction risk assessments across species and regions. Acknowledging the biases in our analysis, our study further emphasises the need to improve monitoring coverage, which is a widely recognised need and not exclusive to BioTIME data used here.

Secondly, the authors main results are inconsistent with their own claims. As can be seen from Figure 3, for taxa in different regions, nearly all species with increasing or decreasing populations are classified as "Least Concern." This intuitively demonstrates that a species population trend cannot reflect its threatened status. Furthermore, nearly all species with randomly fluctuating or stable populations are also "Least Concern"—this finding is disconnected from the authors’ core argument that "population trends can improve extinction risk prediction."

We have clearly indicated that most species in our analysis are classified as "Least Concern" and that there is little overlap between the species in the IUCN and BioTIME databases. In addition, a main (and new) result that we emphasise is the complex links between temporal dynamics and extinction risk categories. Having said that, if decreasing temporal prevalence was uncoupled from extinction risk, why did the prevalence of the different IUCN categories map in rank order onto the classification of population trends that were independently constructed for these data? On the other hand, this comment seems hard to reconcile with the previous one: above, the Reviewer indicates the assessment criteria A and C – based on species trends – are more complex and would render our results “already known”, while here the Reviewer states that “a species population trend cannot reflect its threatened status”. Apologies if there is any misunderstanding in our reading of your comments – but there is no inconsistency with our claims, as all of these estimates and interpretations point to the same conclusion and support our findings. The IUCN extinction risk classification is strongly anchored in five different criteria reflecting abundance and distribution features. This classification is independent of our temporal prevalence dynamics categories, as well as of the monitoring data used in our analysis, which is not designed to assess extinction risk. Given this and since most high-risk species from the IUCN classification have small geographic ranges and/or small populations, it is striking that we are still able to detect a consistent association between decreasing temporal prevalence and increasing extinction risk (and vice versa for increasing temporal prevalence). This strongly supports the relevance of our analysis and the potential to leverage assemblage-level data to provide additional and complementary quantitative analyses to inform conservation efforts.

On the other hand, the authors research results may be subject to survivorship bias. It can also be observed that the majority of taxa studied by the authors are "Least Concern" species. This proportion may be as high as 95% among terrestrial species. Such sampling bias could lead to the authors results primarily relying on differences between "Least Concern" and "Near Threatened/Vulnerable" species, while lacking valid verification for high-risk species.

We appreciate the Reviewer's point, and while we do not explicitly test for sampling biases between the risk categories, our revised sensitivity analysis (see our response below) suggests this is not the case. We have re-run the analysis using a binary threat classification of “non-threatened” = [Least Concern, Lower Risk/Least Concern, Near Threatened, Lower Risk/Near Threatened] vs “threatened” = [Vulnerable, Endangered, Critically Endangered], where our results remain the same as before. Additionally, we clearly acknowledge that most species in our data are classified as “Least Concern”, and have now added that, unlike many IUCN species assessments, assemblage-level data do not specifically focus on threatened species (lines 292-293). This is exactly why we argue that (1) declining trends estimated from assemblage-level data are particularly informative and can be used as early-warning signs, and (2) leveraging assemblage-level data can help alleviate lack of data for less studied taxa and regions.

The authors main analysis focuses on 973 species; however, there is a significant imbalance in geographic and taxonomic coverage. For instance, the study concentrates on Europe and North America, while other regions—tropical areas in particular—are not included. This bias means the conclusions may only apply to temperate vertebrates and cannot be extended to tropical ecosystems or more vulnerable taxa like invertebrates, significantly limiting the study’s global conservation relevance.

The Reviewer is of course correct that there are spatial and taxonomic biases in our data - in particular, our analysis does not include tropical or amphibian data. We believe this point was already addressed to the best of our ability in the previous round. We clearly state these biases throughout the manuscript (e.g., in the abstract, lines 45-46 - “mostly from temperate regions” and lines 293-295 “Notably, amphibian and tropical species were not included in our analysis, two groups that systematically have high extinction risk and decreasing populations.”). We also ran the analyses for temperate regions only, which represented most of our data and still included 52,893 populations of 944 species spanning several terrestrial and marine habitats.

Unfortunately, geographic and taxonomic biases are pervasive across ecological and biodiversity monitoring data. This is also the case for the IUCN, which is biased towards terrestrial and vertebrate species, and surely no one can question its massive importance for global conservation efforts. To this point, ecologists do not (and should not) discard insights from long-term field experiments on biodiversity-ecosystem function because most of them have been conducted in grasslands and forests of Europe and North America. We agree that in an ideal world, we would have systematic spatial and temporal coverage across taxa. As this is not possible, we must make the best use of the data we have, while emphasising the

need for improved monitoring for underrepresented areas and taxa, which tend to be the most diverse. We would be glad to include any further revisions to the text that would better satisfy the Reviewer in addressing this perennial issue.

Additionally, I still hold the view that population research at the regional scale cannot represent the overall situation. In their response, the authors stated that the IUCN also conducts regional-scale endangered risk assessments. However, for many species with wide regional distributions, regional-scale assessment results may be invalid or even contradictory to the overall situation. For example, some species are generally safe at the global level but show a declining trend in a specific region. We cannot directly combine these two sets of results (regional trends and global status).

We completely agree with this point and did not intend to suggest that regional assessments are a substitute for global status classifications. Our reference to the growing number of IUCN regional assessments was precisely intended to highlight the importance of scale when conducting such assessments, which is mentioned in the manuscript (lines 319-322). Importantly, our results show that there is indeed a mapping of local temporal occupancy trends with the IUCN global classification categories. This is important and has never been shown before. As we concluded: "Our results suggest that directional changes in species local prevalence could be harbingers of future changes in global threat status, thus aiding conservation assessments by leveraging assemblage monitoring data." On the other hand, the non-unidirectional links between temporal dynamics and extinction risk are also a key result, highlighting e.g. that non-threatened species have declining trends, while increasing trends for threatened species provide key insights for targeting conservation efforts where they may be most likely to succeed.

In the Methods section, the authors stated: "To ensure that our results were not overly influenced by the predominance of 'Least Concern' species, we further simplified the extinction risk categories into only two groups: 'low risk' (corresponding to 1 in the above classification) and 'high risk', combining the remaining categories (2 to 4)." However, I believe that classifying "Near Threatened" species as "high-risk" constitutes a misunderstanding of IUCN guidelines.

Thank you for pointing this out and compelling us to revise the threat classification for the sensitivity analysis. We have now rerun our analyses with a binary threat classification of "non-threatened" = [Least Concern, Lower Risk/Least Concern, Near Threatened, Lower Risk/Near Threatened] vs "threatened" = [Vulnerable, Endangered, Critically Endangered], (lines 430-433) and have updated all the relevant figures and tables in the SI. The results remain the same as before.

Should the authors consider using changes in population abundance, rather than the presence or absence of a species within a community, as the criterion for determining population trends?

While we appreciate the Reviewer's suggestion, we implemented the categories of temporal prevalence dynamics as a way to deal with highly noisy abundance records and high variability among species. We also note that several studies have focused on abundance trends from assemblage-level time series data (e.g., [5,7]).

**Reviewer #2 (Remarks to the Author):**

The authors have satisfied my previous, minor suggestions and I highly recommend the manuscript for publication in nature Communications. I thank the authors for a pleasant review and wish them all the best.

Thank you very much for your insightful comments and suggestions that helped us improve our previous version.

## References


1 Blowes, S. A. *et al.* The geography of biodiversity change in marine and terrestrial assemblages. *Science* **366**, 339-345, doi:10.1126/science.aaw1620 (2019).

2 Dornelas, M. *et al.* Assemblage Time Series Reveal Biodiversity Change but Not Systematic Loss. *Science* **344**, 296-299, doi:10.1126/science.1248484 (2014).

3 Pilotto, F. *et al.* Meta-analysis of multidecadal biodiversity trends in Europe. *Nature Communications* **11**, 3486, doi:10.1038/s41467-020-17171-y (2020).

4 Crossley, M. S. *et al.* No net insect abundance and diversity declines across US Long Term Ecological Research sites. *Nature Ecology & Evolution* **4**, 1368-1376, doi:10.1038/s41559-020-1269-4 (2020).

5 Crossley, M. S. *et al.* Recent climate change is creating hotspots of butterfly increase and decline across North America. *Global Change Biology* **27**, 2702-2714, doi:https://doi.org/10.1111/gcb.15582 (2021).

6 Daskalova, G. N. *et al.* Landscape-scale forest loss as a catalyst of population and biodiversity change. *Science* **368**, 1341-1347, doi:10.1126/science.aba1289 (2020).

7 Dornelas, M. *et al.* A balance of winners and losers in the Anthropocene. *Ecology Letters* **22**, 847-854, doi:10.1111/ele.13242 (2019).

8 Fraixedas, S., Roslin, T., Antao, L., Pöyry, J. & Laine, A.-L. Opinion: Nationally reported metrics can't adequately guide transformative change in biodiversity policy. *Proceedings of the National Academy of Sciences* **119**, e2117299119, doi:10.1073/pnas.2117299119 (2022).

9 Finn, C., Grattarola, F. & Pincheira-Donoso, D. More losers than winners: investigating Anthropocene defaunation through the diversity of population trends. *Biological Reviews* **98**, 1732-1748, doi:https://doi.org/10.1111/brv.12974 (2023).

10 Boennec, M., Dakos, V. & Devictor, V. Non-linearity and Temporal Variability Are Overlooked Components of Global Vertebrate Population Dynamics. *Diversity and Distributions* **30**, e13932 (2024).

11 d'Eon-Eggertson, F., Dulvy, N. K. & Peterman, R. M. Reliable identification of declining populations in an uncertain world. *Conservation Letters* **8**, 86-96 (2015).

12 Fox, R. *et al.* Insect population trends and the IUCN Red List process. *Journal of Insect Conservation* **23**, 269-278 (2019).

13 Cazalis, V. *et al.* Bridging the research-implementation gap in IUCN Red List assessments. *Trends in Ecology & Evolution* **37**, 359-370, doi:https://doi.org/10.1016/j.tree.2021.12.002 (2022).

14 Edgar, G. J. IUCN Red List criteria fail to recognise most threatened and extinct species. *Biological Conservation* **301**, 110880 (2025).
